# Coupled experimental and numerical parametric study of projectile penetration into granular sandy soils under low-velocity impact

**DOI:** 10.1038/s41598-026-51342-z

**Published:** 2026-06-11

**Authors:** Abdul-Rahman K. Osman, Ahmed Elshesheny, Mohamed S. Zahran, Nabil M. Nagy

**Affiliations:** Civil Engineering Department, Military Technical College, Cairo, Egypt

**Keywords:** Soil-projectile interaction, Low-velocity penetration, Low-velocity impact, Crater formation, Projectile penetration, Finite element modeling, Coupled Eulerian–Lagrangian (CEL), Engineering, Environmental sciences, Natural hazards, Solid Earth sciences

## Abstract

This study presents a parametric investigation of soil-projectile interaction under low-velocity penetration with a focus on the joint effect of granular soil properties and projectile characteristics. First, experimental free-fall vertical drop tests were performed on conical-headed projectiles with different apex angles and masses penetrating compacted sandy soil with different degrees of relative density. More extensive studies were carried out through numerical simulation performed with the Abaqus 2024 software to validate the experimental test results and to examine other variables such as different internal friction angles, dropping heights, projectile diameters, nose configurations, angles of impact, as well as cratering. Complementary analyses were performed to investigate projectile acceleration, energy absorption, stress, and plastic strain distribution in the soil body. The results showed that penetration depth decreased with increasing sand relative density, internal friction angle, projectile diameter, and projectile apex angle, while it increased with increasing projectile mass, drop height, and impact angle. Crater diameter was found to increase mainly by increasing drop height and projectile diameter, while its sensitivity to internal friction angle remained comparatively limited within the investigated range. To improve the physical interpretability of the results, dimensionless correlations were developed and combined with correlation-based and response-span-based sensitivity assessments to clarify the relative importance of the governing parameters. Within the investigated conditions, the study provides a validated experimental–numerical framework for interpreting low-velocity projectile penetration in granular sandy soils and for identifying the parameters that most strongly control penetration depth and crater development.

## Introduction

Designing protective structures capable of defending projectile penetration requires a comprehensive understanding of soil-projectile interaction mechanisms across different velocity regimes and geomaterial types. For buried and partially buried facilities in arid and semi-arid regions, defensive capacity optimization depends upon two complementary objectives: systematic characterization of projectile penetration mechanisms in granular media and development of effective countermeasures to mitigate penetration threats. Many countries depend on buried or partially buried protective structures in granular-soil environments, where experimental and numerical investigations have consistently demonstrated that sandy soils provide superior mechanical resistance to projectile penetration compared with cohesive materials^1–3^. This enhanced resistance derives from the mechanical response of granular materials under impact: compaction of sandy soil to elevated relative densities reduces void spacing, increases frictional mobilization, and enhances energy dissipation, thereby augmenting shear strength and reducing projectile penetration depth^4–6^.

Systematic investigation of penetration mechanisms requires examination of soil-projectile interaction across a spectrum of impact velocities and loading conditions. Extensive research has characterized projectile penetration into geomaterials across a broad spectrum of impact velocities, with established classification frameworks organizing this diversity into mechanistically distinct regimes. Velocity regimes are classified relevant to penetration mechanics into five main categories^7–11^. The low-velocity regime (0–25 m/s) encompasses gravitationally driven or quasi-static loading, producing penetration through simple embedment without significant material disruption. The sub-ordnance regime (25–500 m/s) is typically accessed through gas gun experimentation, where kinetic energy dominates but catastrophic material failure remains limited. The nominal ordnance regime (500–1300 m/s) encompasses conventional ballistic impacts, wherein both projectile and target generally maintain structural integrity. Ultra-ordnance (1300–3000 m/s) and hypervelocity (> 3000 m/s) regimes introduce severe material response: projectiles may fragment or vaporize, and targets may experience melting and intense strain-rate effects. Within this velocity framework, systematic investigation of soil-projectile interaction has expanded across multiple geomaterial types and loading conditions.

Research spanning low-velocity penetration^12–17^ through high-velocity impact scenarios^18–21^ has characterized the behavior of diverse soil types including clays, saturated sands, dry sands, and rock formations—demonstrating that geomaterial type significantly influences penetration resistance and crater morphology^12,22–31^. In parallel, investigations have established the critical influence of projectile parameters on penetration performance. Projectile characterization such as mass and nose apex angle has been shown to substantially affect penetration depth and crater development, with sharper geometries and increased mass generally enhancing penetration efficiency^16,17,32–34^. Significant efforts have also been dedicated to investigating projectile impact on various defensive materials such as metals and composite materials to improve the efficacy of armor systems and protective components^35–38^.

**Fig. 1:**
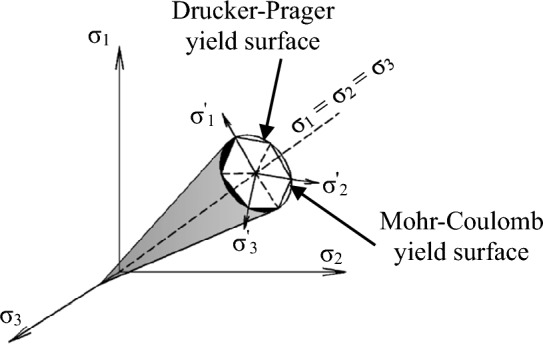
Comparison of Drucker-Prager and Mohr–Coulomb yield criteria in principal stress space.

Advancements in numerical modeling now enable high-fidelity simulations to augment research, thereby decreasing time, cost, and experimental complexity, while facilitating the exploration of penetration scenarios that are challenging to replicate physically. In this context, advanced methodologies such as the coupled Eulerian–Lagrangian (*CEL*) approach^39,40^ and smoothed particle hydrodynamics (*SPH*)^28,41,42^ are widely used to represent various soil–projectile interaction scenarios. Moreover, constitutive modeling of geomaterials has facilitated a more precise modelling of sandy soil behavior under various loading conditions. The Mohr–Coulomb model (*MC*) remains the traditional selection in soil mechanics for simulating shear failure, assuming that failure occurs when shear stress reaches a threshold that varies linearly with the internal friction angle (*ϕ*) and cohesion (*c*); it is extensively adopted for modeling sandy soils at low penetration velocities due to its ability to capture shear strength as observed in triaxial and direct shear tests^9,43–48^. Conversely, the Drucker-Prager model (*DP*) offers a smooth yield surface in principal stress space, determined by mean stress and octahedral shear stress, thereby enhancing numerical stability in dynamic simulations characterized by high strain rates, such as high-velocity projectile impacts and blast loading in sandy soils^49,50^ (Fig. 1).

Despite of all this extensive research, an important scientific gap still remains, as representative prior studies on sand penetration have mainly focused on selected variables, specific mechanisms, or particular response measurements rather than focusing on a unified comparison of the relative importance of the governing parameters ^15,31,32^. Although the importance of identifying the most influential impact parameters has been increasingly recognized in the broader impact and penetration literature, such efforts have been more explicitly developed in materials other than soil, including laminated composite plates^51^, thin concrete slabs^52^, and cement-based materials^53^. By contrast, comparable systematic ranking of the governing parameters remains relatively limited in the low-velocity penetration literature for granular sandy soils, particularly within a unified experimental–numerical framework^54^.

To address this gap, the present study introduces a comprehensive parametric investigation of low-velocity projectile penetration in granular sandy soils, utilizing experimental and numerical methodologies to examine the influence of diverse variables encompassing both soil properties and projectile characteristics within a unified framework. The study also introduces a structured interpretative framework based on Spearman correlation heatmapping, and response-span-based sensitivity ranking. This additional layer of analysis is used to quantify the comparative influence of the investigated parameters, identify the most critical factors controlling penetration depth and crater diameter, and transform the results from a set of isolated parametric observations into a more systematic sensitivity assessment.

This investigation starts with an experimental phase employing vertical free-fall drop tests of four metallic conical-nosed projectiles with different apex angles ($\alpha$) and masses (*m*_*p*_). Projectiles are released to free-fall onto mechanically compacted granular sand, without drying or moisture addition, at varied relative densities (*D*_*r*_). This experimental configuration permits systematic examination of the influences of *D*_*r*_, $\alpha$, and *m*_*p*_ on penetration depth (*d*_*p*_), while simultaneously providing comprehensive data for numerical model validation. Following the experimental phase, in-depth numerical analysis is conducted using Abaqus Explicit Dynamics 2024. Initially, four validation models were developed to compare numerical predictions with laboratory measurements, confirming that the numerical approach accurately represents penetration behavior. In these models, soil is represented as an Eulerian domain while projectiles are modeled as discrete rigid bodies with a reference point at the nose tip, permitting detailed investigation of projectile motion and deceleration during penetration. Following model validation, numerical analysis is substantially expanded to enhance the investigation of $\alpha$, and *m*_*p*_ influences penetration depth through inclusion of additional apex angles and greater projectile mass.

The numerical investigation subsequently examines the influence of soil internal friction angle (*ϕ*), drop height (*H*), projectile diameter (*D*), and different projectile nose geometries—including conical, ogival, parabolic, hemispherical, and blunt configurations—as well as the effect of oblique projectile impact angles (*θ*). Each configuration is systematically examined for *d*_*p*_, crater diameter (*D*_*c*_), energy dissipation (*PE*_*d*_), projectile deceleration, and stress–strain distribution in surrounding soil. To further strengthen the interpretation of the results, the study also employs dimensionless analysis together with Spearman correlation mapping and response-span-based sensitivity ranking to quantify the comparative influence of the governing parameters, identify the most critical factors controlling penetration depth and crater development within the investigated conditions, and convert the parametric results into a more systematic sensitivity framework within the investigated conditions. The following sections present experimental and numerical methodologies in detail, followed by comprehensive discussion of results and analyses, culminating in thorough understanding of sandy soil behavior under low-velocity projectile penetration.

## Experimental work

**Fig. 2:**
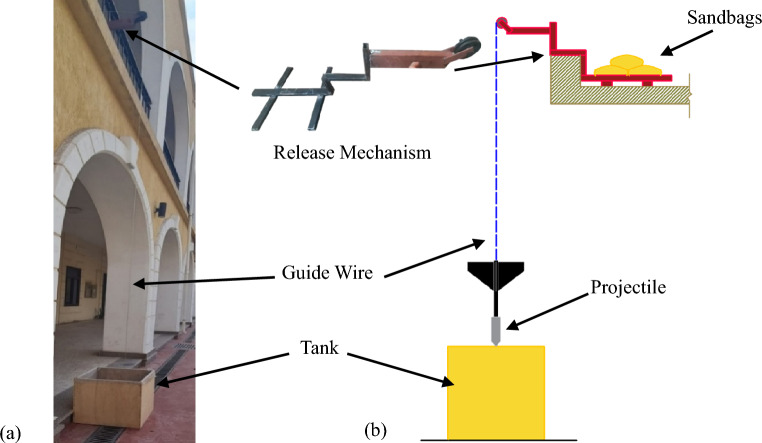
(**a**) Physical experimental setup for projectile penetration tests; (**b**) schematic diagram of the setup components.

A series of laboratory-scale experimental tests were conducted to investigate the influence of key initial parameters on the penetration of projectiles into sandy soil and to provide reliable data for validating current and future numerical simulations. All observed effects on penetration depth were captured and analyzed, and the detailed experimental methodology and results are presented and discussed in the next sections. An overall experimental setup has been developed, as shown in Fig. 2, which is allowing conducting of low-velocity free fall penetration experiments on compacted layers of sandy soil, that would represent a controlled physical representation for understanding low-velocity penetration mechanisms in sandy soils, and ensuring controlled repeatable scenarios of projectile impact against the ground target.

The experimental setup consists of a specially designed release mechanism which allows releasing the projectile at predetermined time, after ensuring the stability of the projectile. The release mechanism was manufactured using rigid rectangular hollow steel sections with cross-sectional dimensions of 5 cm × 3 cm, which were welded together to enable controlled release of the projectile in accordance with the geometry of the building from which the projectile was released. To stabilize the release mechanism and keep it in a fixed position without any relative movement, several heavy sandbags were installed. A guide wire of 1 mm diameter was used to attach the projectile with the releasing mechanism. To ensure that the projectile is precisely aligned with the center of the testing tank, it was initially placed such that it merely contacts the center of the testing tank, with the longitudinal axis aligned with the center of container in all experiments. The guide wire is then pulled gently backwards to raise the projectile to the predetermined drop height (*H* = 4.295 m), considering its exact vertical alignment and stability. Finally, a scissor is used to cut the wire and release the projectile, enabling its vertical free fall.

### Materials

#### Sand

Natural coarse sand was used to prepare the homogeneous testing target representing the ground. The physical and mechanical properties of the sand summarized in Table 1 were obtained through a series of laboratory tests. The sand was utilized in its original state, without drying or adding water.

**Table 1:** Properties of the Sand.

Test	Description	Value
Sieve analysis	Uniformity Coefficient, *C*_*u*_	3.0
	Coefficient of Curvature, *C*_*c*_	0.94
	Effective grain size, *D*_*10*_ (mm)	0.28
	*D*_*30*_ (mm)	0.48
	*D*_*60*_ (mm)	0.85
Compaction	Optimum Moisture Content, *OMC* (%)	8.7
	Maximum dry unit mass, ${\rho}_{d,max}$ (gm/cm^3^)	1.8
	Minimum dry unit mass, ${\rho}_{d,min}$ (gm/cm^3^)	1.48
	Maximum void ratio, *e*_*max*_	0.753
	Minimum void ratio, *e*_*min*_	0.444
	Specific Gravity, *G*_*s*_	2.6
	Relative Density, *D*_*r*_ (%)	95
	Actual unit mass of sand, ${\rho}_{Act}$ (gm/cm^3^)	1.781
Shear box and Triaxial	Peak angle of internal friction, *ϕ*_*peak*_ (°)	36.7
	Critical angle of internal friction, *ϕ*_*critical*_ (°)	33.8
	Dilation angle, $\psi$ (°)	2.9°
	Cohesion, *c* (MPa)	0.0
	Young’s Modulus of elasticity, *E* (MPa)	50
	Poisson’s ratio, *v*	0.3

Where; $\psi$ = *ϕ*_*peak*_–*ϕ*_*critical*_^55^.

#### Projectiles

**Fig. 3:**
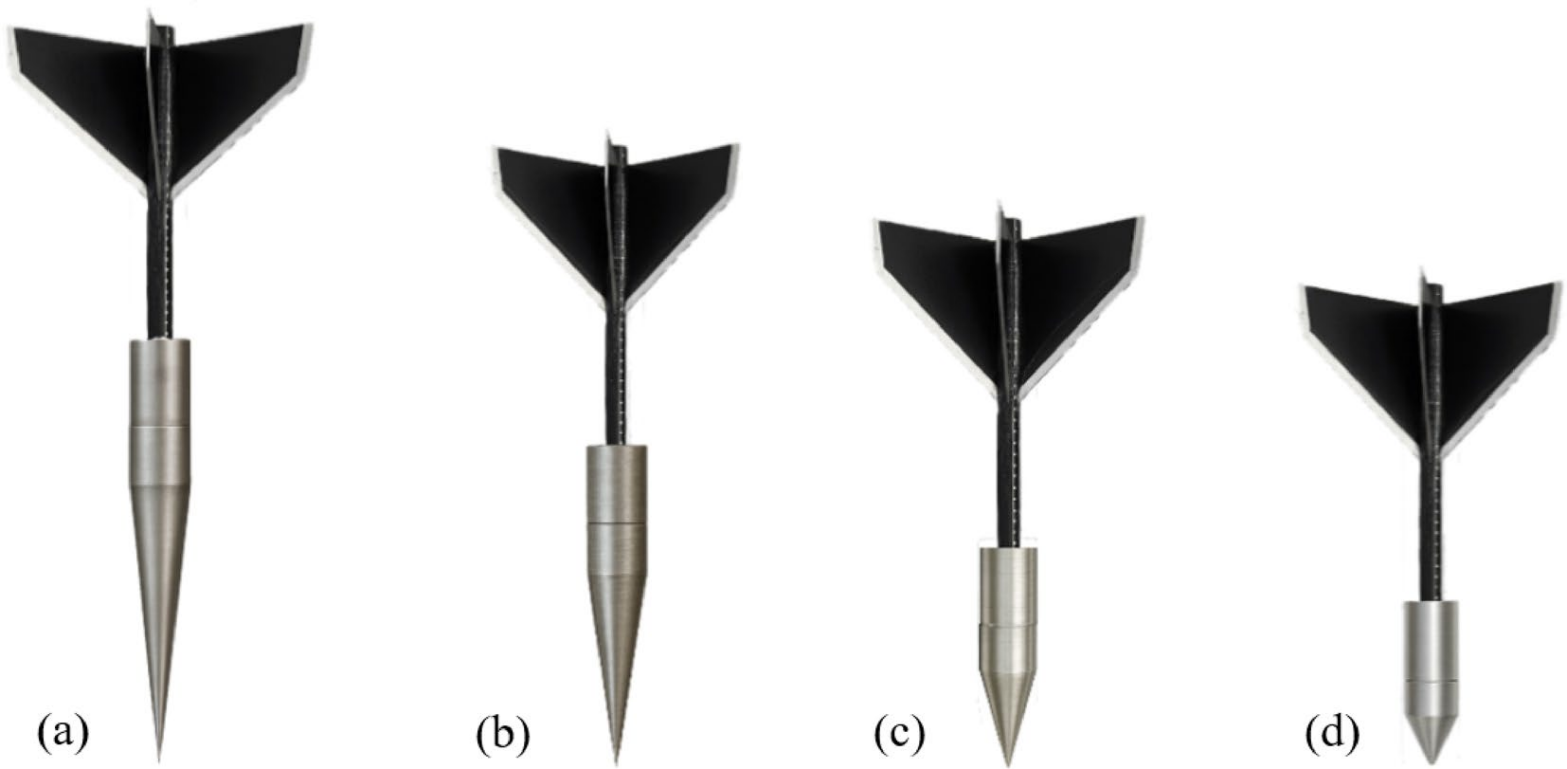
Steel projectiles with conical noses of different apex angles: (**a**) 10°, (**b**) 30°, (**c**) 60°, and (**d**) 90°.

Rigid steel projectiles with a diameter of 50 mm, were manufactured using CNC machining to produce conical noses with four distinct apex angles: 10°, 30°, 60°, and 90° as illustrated in Fig. 3.

**Fig. 4:**
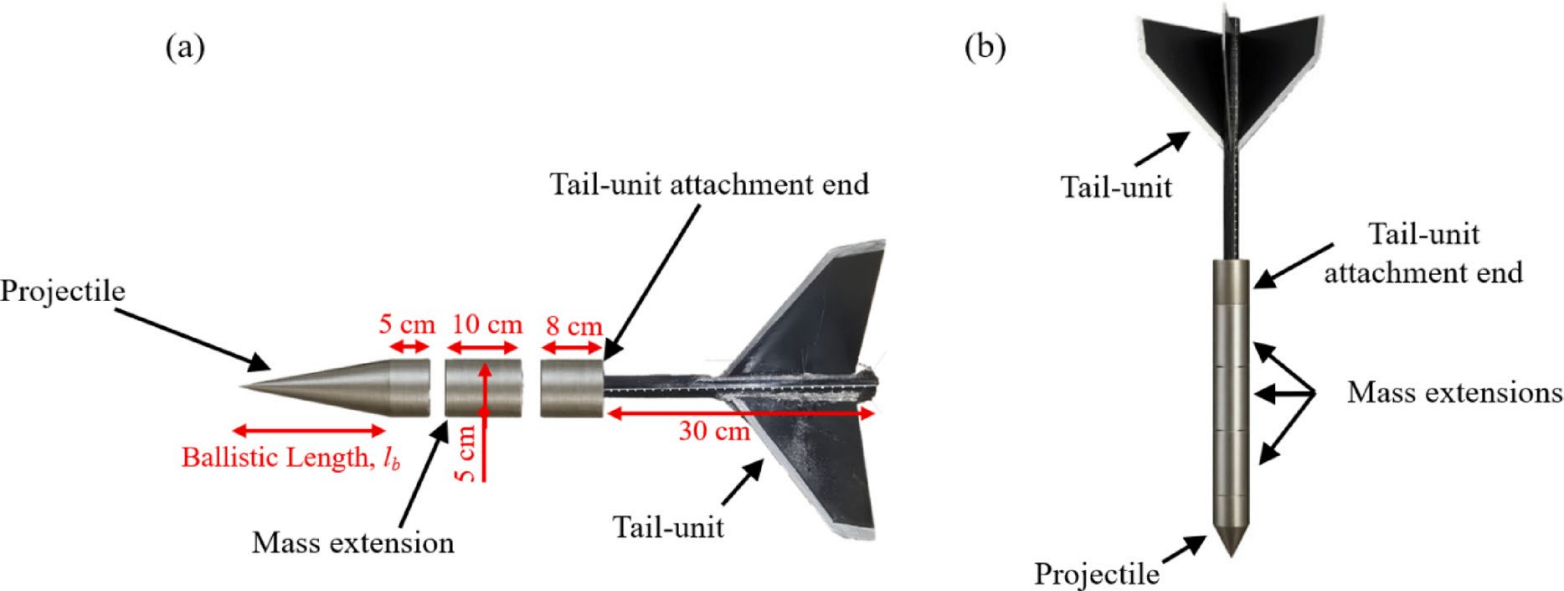
Projectile system configuration: (**a**) disassembled view, (**b**) fully assembled.

The selected angles cover a wide range of conical noses, which makes it possible to systematically study how conical nose apex angle affects penetration behavior. To manage and modify the mass of the projectile, removable mass extensions were produced. Each extension provides about 1.467 kg, which allows changing the entire mass of the projectile despite changing the nose apex angle. A plastic tail unit was produced attached with steel end at the rear of the projectile to make it more stable during free fall and to keep it from tilting or impacting at an angle of inclination. The tail-unit with the steel end has fixed total mass of 1.685 kg. The adaptable structure allows assembly and disassembly of the projectile, mass extensions, and tail unit for any chosen nose angle. Figure 4 shows the assembled and disassembled overall configuration, dimensions, and removable parts of the projectile system.

#### Testing tank and measuring technique

**Fig. 5:**
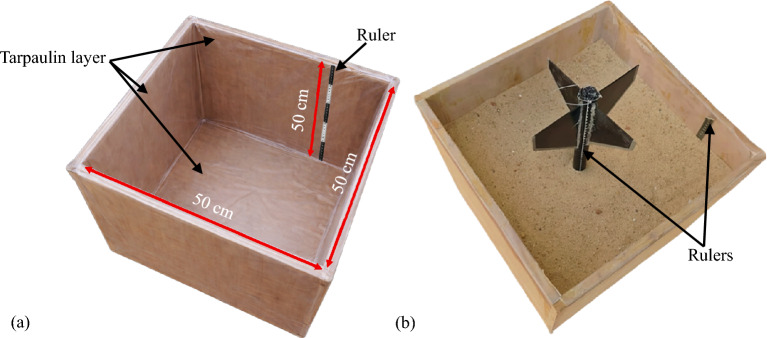
(**a**) Lined wooden test tank; (**b**) measurement rulers on projectile and test tank.

Hardwood of 18 mm thickness was used to construct a rigid testing tank with an internal square cross-section of 50 cm × 50 cm and a height of 50 cm shown in Fig. 5. To avoid the boundary condition effects, the tank dimensions were selected in accordance with established guidelines, such that the ratio between the side length (*B*) and projectile diameter ($D$), shouldn’t be less^56^ than five ($B/D\ge 5$). Accordingly, the selected tank and projectile dimensions provided a ratio of $B/D=10$. All internal walls of the testing tank were lined with a tarpaulin layer prior to sand placement to reduce friction between the sand particles and the hardwood surface. A ruler was attached to the tank’s inner wall to measure the height of the poured sand. Another ruler was attached to the tail-unit rod of the projectile to measure its penetration depth within the soil.

#### Experimental testing program

A total of 15 drop tests were conducted, as summarized in Table 2, including three repeated tests of one reference case to confirm repeatability; all remaining cases were tested one time under identical environmental and boundary conditions. Four projectile apex angles, $\alpha$ of 10°, 30°, 60°, and 90° were used. The effect of different original projectile mass was initially neglected because the total mass varied only slightly among these projectiles. All projectiles were released in free-fall condition from a fixed height of 4.295 m, which was the maximum available height in the laboratory and corresponds to an impact velocity of approximately 9.18 m/s, which was calculated using Eq. (1).

1 $$\mathrm{v}= \sqrt{2gH}$$

where (v) is the projectile’s impact velocity, and (*g*) is the gravitational acceleration.

**Table 2:** Experimental test scheme.

Test No	Variable	Test configurations			
		*H* (m)	*D*_*r*_ (%)	$\alpha$(°)	*m*_*p*_ (kg)
T1–T3	Repeatability tests	4.295	95	30	2.76
T4–T7	Sand relative density, *D*_*r*_ (%)	4.295	25	90	2.445
			55		
			75		
			95		
T8–T11	Projectile apex angle, $\alpha$ (°)	4.295	95	90	effect neglected
				60	
				30	
				10	
T12–T15	Projectile mass, *m*_*p*_ (Kg)	4.295	95	90	2.445
					3.87
					5.33
					6.81

Influence of varying the relative density of the sand was investigated, while maintaining constant apex angle, mass, and drop height. The *D*_*r*_ values was 25%, 55%, 75%, and 95%, respectively. For analyzing the effect of conical projectile nose apex angle, $\alpha$ (10°, 30°, 60°, and 90°), the constant parameter was *D*_*r*_ = 95%, *H* = 4.295 m, and the original variations in projectile mass due to different nose apex angles were ignored because the difference in masses were slight. Finally, for projectile mass effect, the constant parameters were $\alpha$ = 90°, *D*_*r*_ = 95%, and *H* = 4.295 m, while attachable mass extensions were added to obtain *m*_*p*_ of 2.445 kg, 3.87 kg, 5.33 kg, and 6.81 kg.

## Numerical modeling

A three-dimensional numerical framework is employed to simulate projectile penetration into sandy soil using the coupled Eulerian–Lagrangian (*CEL*) formulation implemented in Abaqus 2024. The numerical study begins with the development of four validation models that replicate experimental cases from the projectile-apex angle study, matching the projectile geometry, drop height, and corresponding soil conditions. Numerically obtained penetration depths are systematically compared with the laboratory measurements, revealing acceptable agreement between numerical and experimental tests, which ranged between 92 and 99%. After this level of agreement is achieved, the validated model is adopted as a basis for a broader parametric investigation. Numerical modeling also enables detailed examination of projectile kinematics and soil response, including velocity–time history, plastic energy dissipation, and distribution of plastic strain and stress, which are deeply discussed in the results and discussion section. The following subsections present the numerical model implementation details.

### Modeling framework

**Fig. 6:**
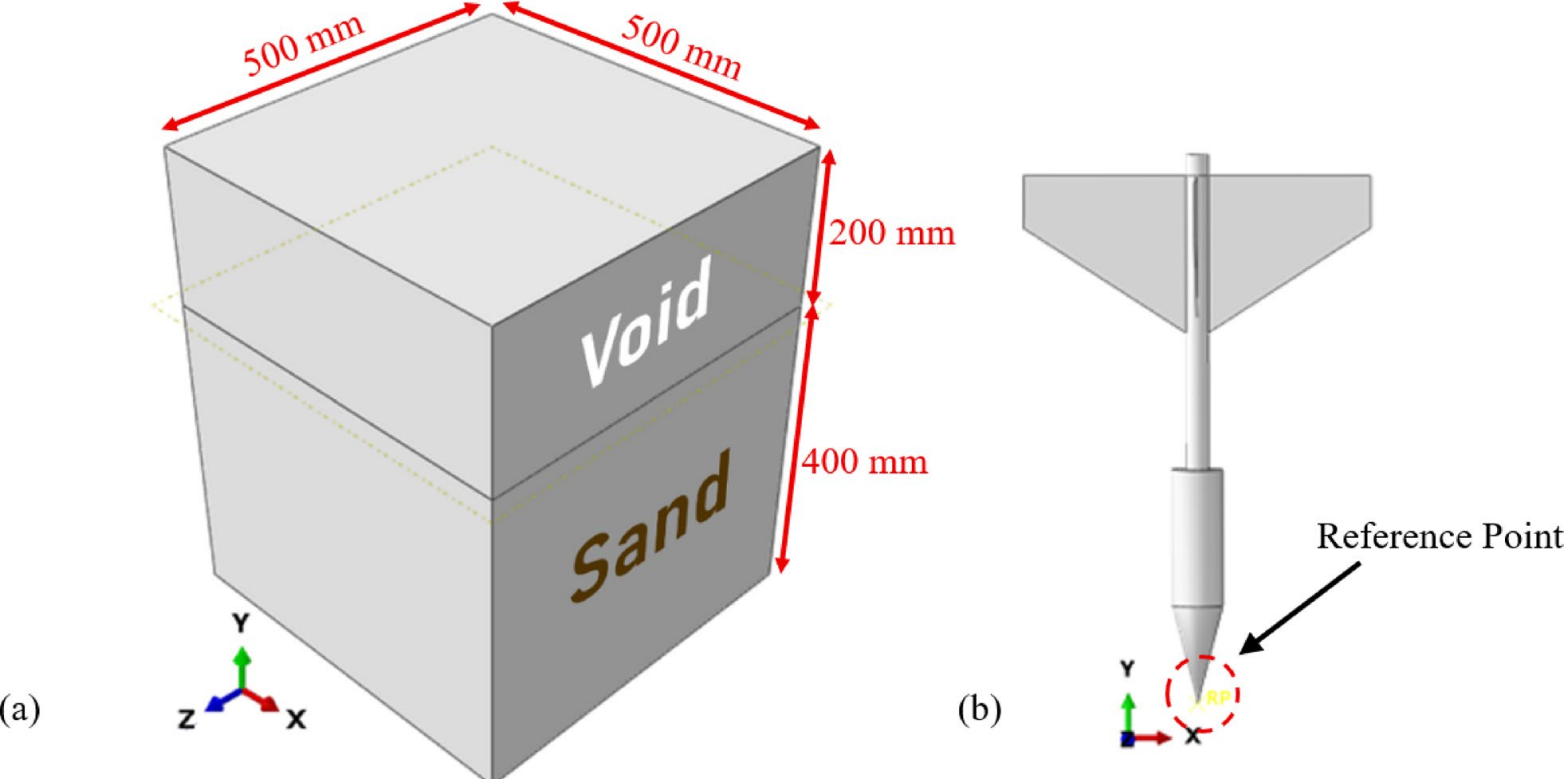
Coupled Eulerian–Lagrangian framework of (**a**) sand as Eulerian domain, (**b**) projectile as discrete rigid part.

A coupled Eulerian–Lagrangian (*CEL*) framework was applied, in which the sand is modeled as an Eulerian part. An additional clearance of 200 mm was introduced above the initial sand surface to allow upward soil displacement during the penetration process and to perform as the free surface for the soil heaving movement^46^. Projectile, represented as a discrete rigid Lagrangian part with a reference point which was defined at the nose tip of each projectile, to which the measured mass and prescribed impact velocity were assigned, thereby accurately representing the projectile’s inertial properties and ensuring realistic coupling between its motion and the sand domain in the *CEL* simulations (Fig. 6). The projectile was initially positioned at the center of the sand surface, aligned along the negative $\mathrm{y}$-axis direction, and assigned an impact velocity computed from the free-fall relationship given in Eq. (1). The sand was modeled in its very dense state, corresponding to *D*_*r*_ = 95%, which was kept constant throughout numerical investigation, because it exhibited the highest penetration resistance in the laboratory tests.

### Soil constitutive model

The sand was modeled using an elasto-plastic Mohr–Coulomb (*MC*) failure criterion (Table 3), which is widely adopted for frictional granular materials subjected to low-velocity projectile penetration^9,43–48^. In Abaqus, the *MC* constitutive model combines linear elastic behavior with an elastoplastic yield condition based on the *MC* criterion, where yielding occurs when the shear stress on a potential failure plane reaches a linear function of the normal stress on that plane^57^. The *MC* yield function is defined as:

2 $$\tau =c+{\sigma}_{n}.\mathrm{tan}\left(\phi \right)$$

where ($\tau$) is the shear stress, (c) is the soil cohesion, (${\sigma}_{n}$) is the normal stress on the failure plane, and is ($\phi$) the internal friction angle.

**Table 3:** Soil input parameters.

Young’s modulus, *E* (Mpa)	Poisson’s ratio, *ν*	Density, ${\rho}_{d}$ (gm/cm^3^)	Friction angle, *ϕ* (°)	Dilation angle, $\psi$ (°)
50	0.3	1.781	36.7	2.9

**Fig. 7:**
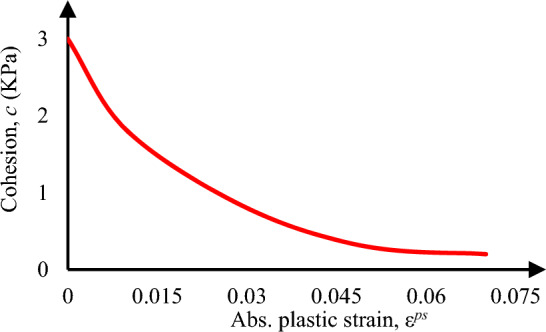
Cohesion softening curve used in the Mohr–Coulomb soil model.

During loading, elastic strains are computed until the yield condition is met, after which plastic flow evolves according to a defined flow rule controlled by the dilation angle. The Abaqus implementation includes isotropic cohesion hardening/softening and allows the definition of tension and compression cutoffs for improved numerical stability and produce more realistic soil response predictions. Although sandy soils are inherently cohesionless materials, a very small apparent cohesion was introduced in the present study together with strain softening, as illustrated in Fig. 7. This apparent cohesion does not represent true interparticle bonding; instead, it was employed as a numerical regularization parameter to control strain localization and to ensure stable solutions in large-deformation *CEL* simulations of penetration problems. Similar numerical treatments have been widely adopted in previous studies on soil–projectile interaction and penetration into granular media^58–62^. The interaction between the projectile and the sand domain was modeled using a general contact formulation, with a friction coefficient of 0.35 assigned between steel-sand interface, consistent with values reported for steel–sand interfaces in the engineering literature^63^.

### Meshing and boundary conditions

**Fig. 8:**
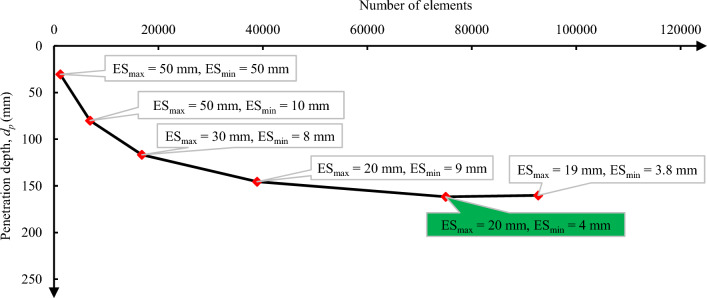
Mesh sensitivity analysis for and selection of the adopted mesh used in the CEL simulations.

A mesh sensitivity analysis for the sand domain was conducted using the validation configuration corresponding to the conical projectile with an apex angle of $\alpha$ = 90°, impacting very dense sand (Dr = 95%), in order to evaluate the influence of mesh refinement on the predicted penetration depth. Several graded meshes were examined by progressively reducing the minimum local element size along the penetration path while maintaining the same overall meshing strategy. The examined meshes were selected such that the element aspect ratio (*AR*) remained at *AR* ≤ 5 as a practical mesh-quality criterion, to achieve a satisfactory element quality during refinement^57,64–66^. As illustrated in Fig. 8, the predicted penetration depth changed markedly from the course meshes and then exhibited only a marginal variation between the two finest discretizations. Accordingly, the mesh with a global element size (*ES*_*max*_) of 20 mm near the boundaries and a minimum local element size (*ES*_*min*_) of 4 mm in the interaction region was adopted for the subsequent simulations, since the finer discretization produced only a negligible change in penetration depth compared with the associated increase in computational cost. The sand domain was discretized into 75,000 elements.

**Fig. 9:**
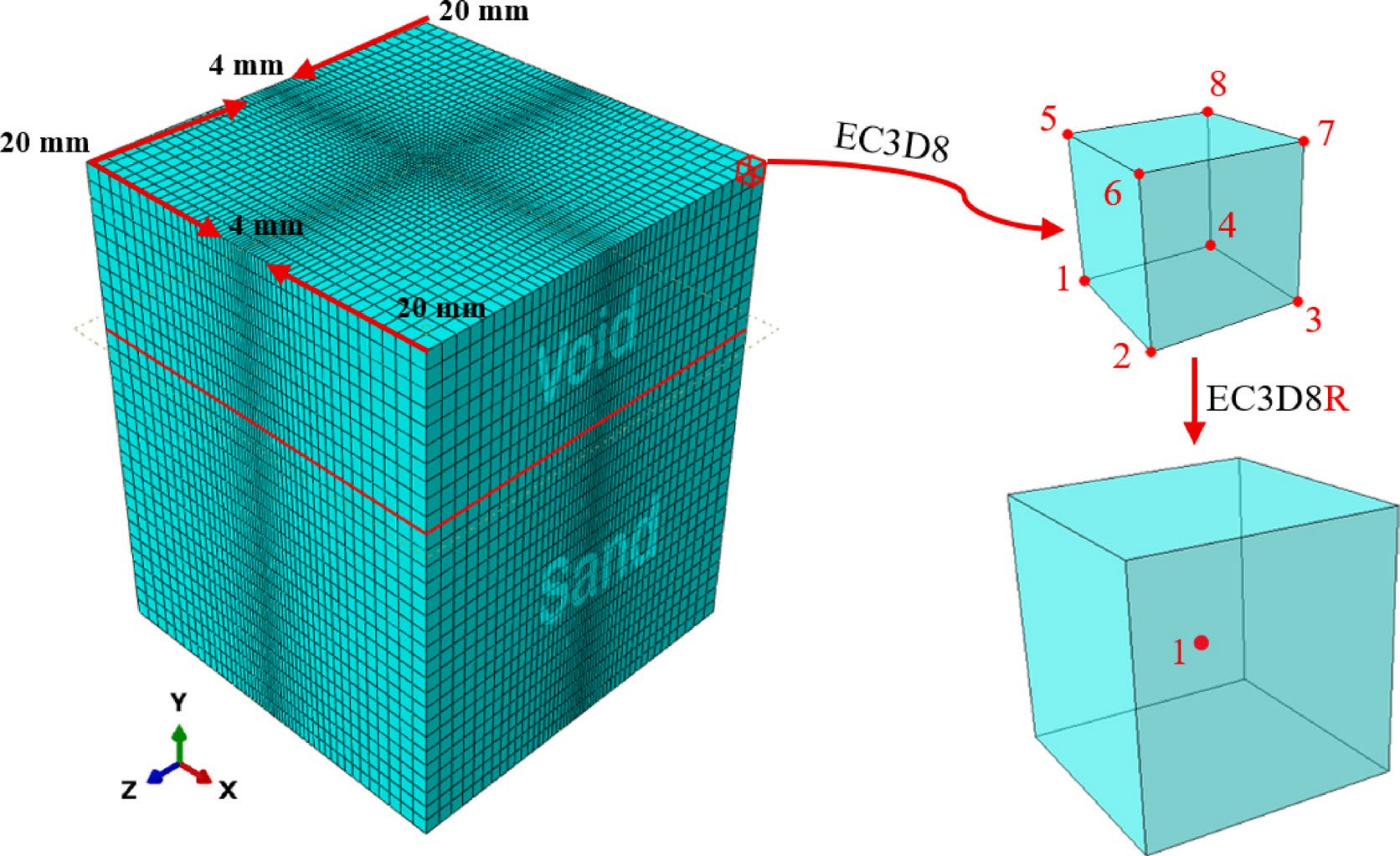
Graded mesh of the sand domain using EC3D8R elements, with refinement near the penetration path and coarser mesh at boundaries.

The sand domain was discretized using structured eight-node Eulerian brick elements with reduced integration (*EC3D8R*). These elements are based on the standard *EC3D8* formulation, in which reduced integration is applied by employing a single integration point, thereby lowering the computational cost and mitigating numerical issues such as volumetric locking, while maintaining robustness in simulations involving large deformations and material flow^57^. A graded meshing strategy was implemented, with coarser elements near the lateral boundaries (20 mm) and a progressively refined mesh in the projectile-soil interaction region, reaching a minimum element size of approximately 4 mm along the penetration path, as shown in Fig. 9.

**Fig. 10:**
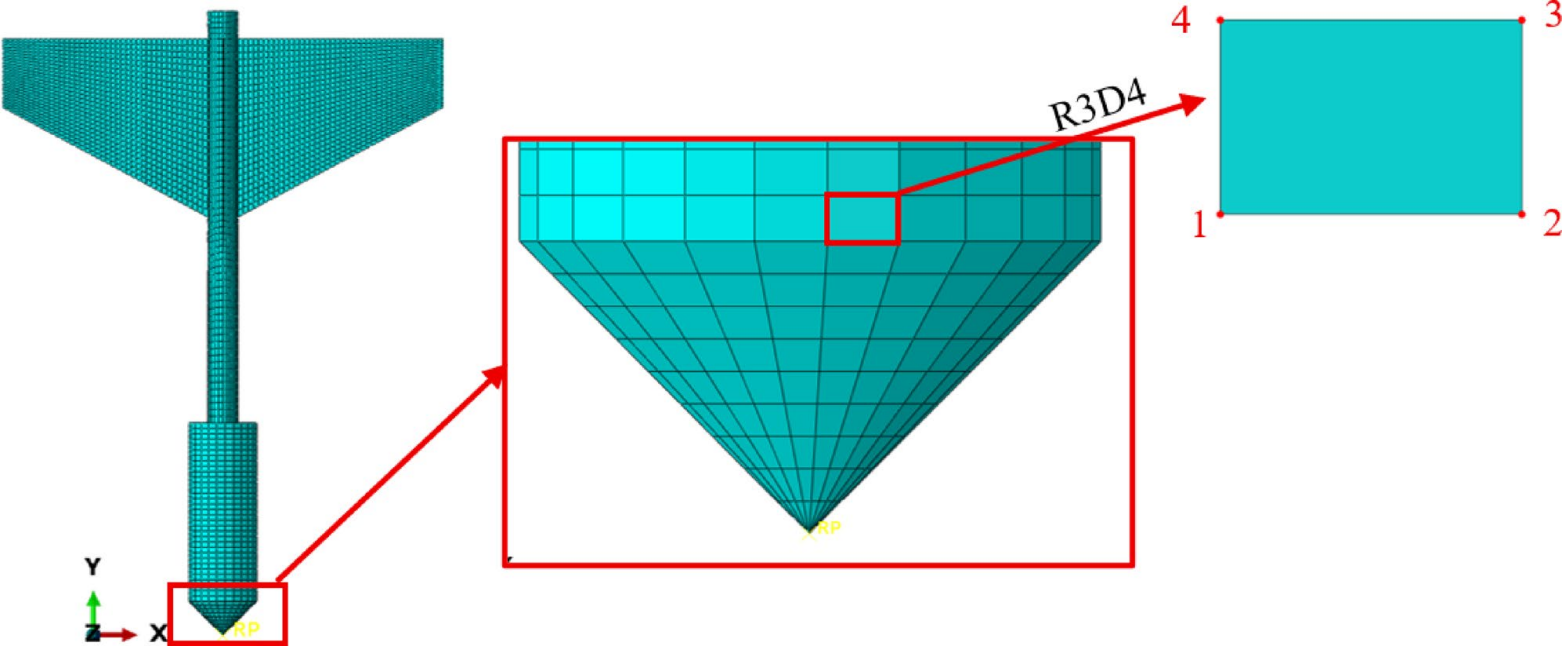
Projectile mesh using R3D4 discrete rigid shell elements with sweep technique.

The projectile was discretized using four-node discrete rigid shell elements (*R3D4*) with an element size of approximately 4 mm to ensure proper compatibility with the surrounding Eulerian soil mesh in the contact region. A sweep meshing technique was employed, which is particularly well suited for conical and cylindrical geometries. The *R3D4* element represents a rigid, four-node quadrilateral shell formulation^57^ in which the projectile is assumed to remain undeformed during impact, thereby reducing computational cost while accurately transferring contact forces to the surrounding medium. The resulting mesh exhibits good element quality without irregular shapes that could adversely affect numerical stability and accuracy, as illustrated in Fig. 10.

**Fig. 11:**
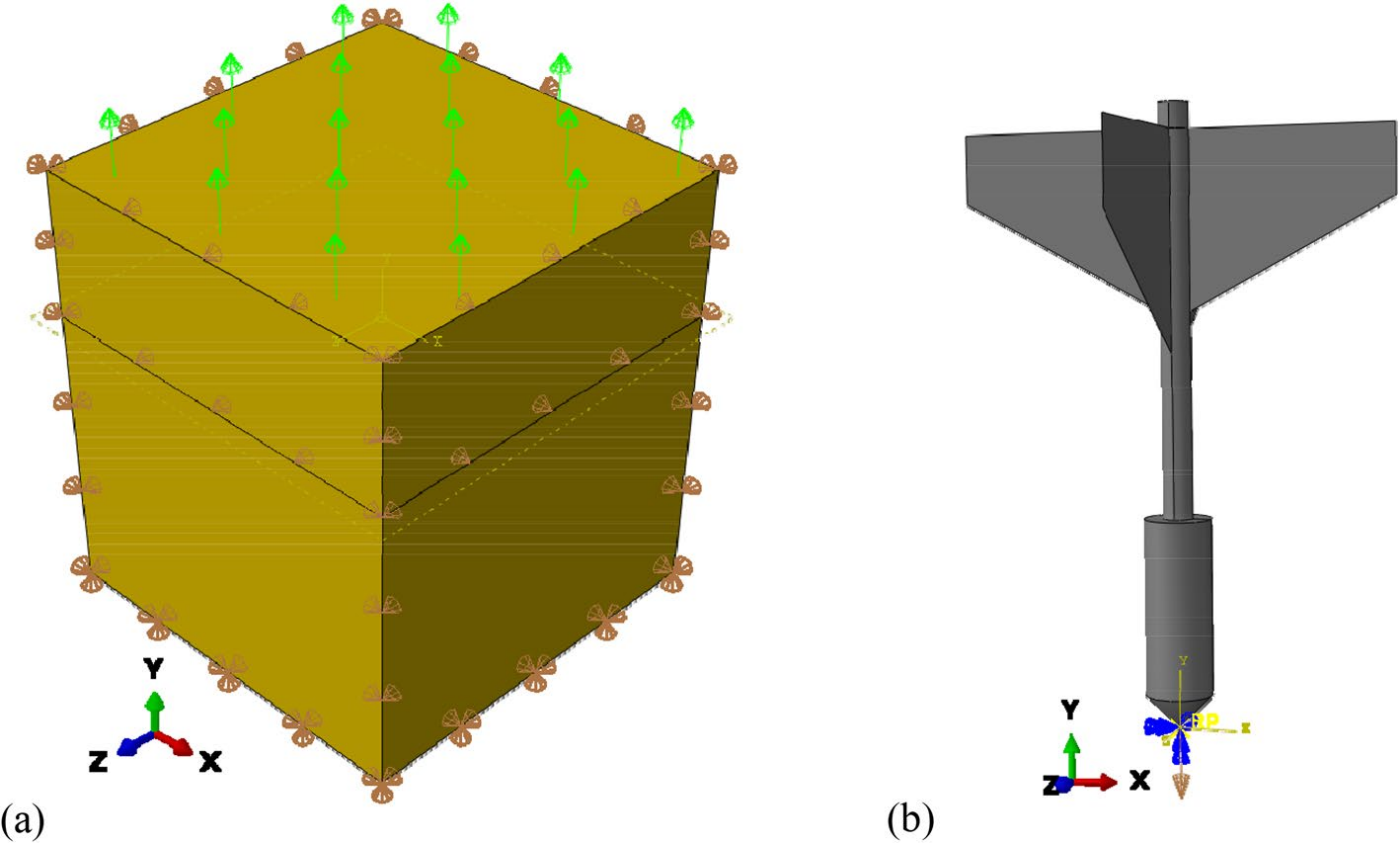
Numerical boundary conditions applied for (**a**) the sand domain, and (**b**) projectile.

Appropriate boundary conditions were applied to accurately represent the physical constraints of the penetration problem. The projectile was constrained at its reference point by fixing all rotational degrees of freedom, ensuring purely translational motion along the penetration direction. For the Eulerian sand domain, the bottom surface was fully constrained in all directions to represent a rigid underlying support. The lateral boundaries were restricted in their normal directions perpendicular to each face. The top surface of the Eulerian domain was defined as a free outflow boundary, permitting material to exit the computational domain during large deformation and material flow induced by projectile penetration. Figure 11 illustrates the applied boundary conditions.

### Numerical testing program

A comprehensive numerical parametric study was carried out to extend the investigation beyond the constraints of laboratory testing and to systematically evaluate the influence of key governing parameters. The numerical program was conducted ensuring that, for each investigated parameter, all remaining parameters were held constant. This controlled approach ensured that observed variations in the results could be directly attributed to the parameter under consideration. Table 4 presents the complete numerical matrix, encompassing variations in projectile nose geometry, apex angle, mass, diameter, drop height, internal friction angle of sand, and impact angle. All simulations were conducted under identical boundary conditions and for a very dense sand state with a relative density of *D*_*r*_ = 95%.

**Table 4:** Numerical test scheme.

Test No.	Variable	Test configurations							
		*H* (m)	*D*_*r*_(%)	ϕ (°)	Nose geometry	α (°)	m_p_ (kg)	*D*(mm)	$\theta$(°)
M1–M4	Projectile apex angle, $\alpha$ (°)	4.295	95	36.7	Conical	105	effect neglected	50	90
						75			
						45			
						20			
M5–M8	Projectile mass, *m*_*p*_(Kg)	4.295	95	36.7	Conical	90	8.265	50	90
							9.72		
							11.175		
							12.63		
M9–M15	Angle of internal friction, *ϕ *(°)	4.295	95	36	Conical	30	2.76	50	90
				37					
				38					
				39					
				40					
				41					
				42					
M16–M19	Drop height, *H* (m)	4.295	95	36.7	Conical	90	2.445	50	90
		10							
		25							
		50							
M20–M23	Projectile diameter, *D* (mm)	4.295	95	36.7	Conical	30	2.76	25	90
								50	
								75	
								100	
M24–M28	Projectile nose geometry	4.295	95	36.7	Conical	30	2.76	50	90
					Ogival (*CRH* = 3.7)				
					Hemispherical				
					Parabolic (*l*_*b*_ = 93 mm)				
					Blunt				
M29–M33	Impact angle, $\theta$ (°)	4.295	95	36.7	Conical	30	2.76	50	90
									80
									60
									45
									30

## Results and discussion

### Experimental results

To provide a unified basis for comparison, both the governing parameters and the corresponding response were expressed in dimensionless form. For the projectile apex angle, the adopted transformation was based on the geometric definition of the conical nose itself. As shown by the trigonometric relation in Eq. (3), the semi-apex angle arises naturally from the right triangle formed by the ballistic length of the conical head, ${l}_{b}$, and the projectile radius, ($D/2$). This makes ($\alpha /2$), rather than ($\alpha$) alone, the directly relevant geometric quantity controlling the cone inclination.

3 $$\mathit{tan}(\alpha )=\frac{D}{2{l}_{b}}$$

On this basis, the dimensionless angular parameter was defined in Eq. (4) as the sharpness index (${\alpha }^{*}$) using the cotangent of the semi-apex angle. This form was selected because it provides a physically meaningful measure of nose slenderness, increasing systematically as the tip becomes sharper and the cone becomes more elongated. By contrast, a direct normalization of the apex angle itself would retain the numerical ordering of the test cases but would not represent the geometric change in sharpness with the same clarity or physical relevance.

4 $${\alpha }^{*}=cot(\alpha /2)$$

The projectile mass was expressed as dimensionless projectile mass parameter (${m}_{p}^{*}$) through Eq. (5), where the normalization term (${\rho}_{d}{D}^{3}$) represents a characteristic mass of the sand associated with the local penetration scale. This form was preferred because it relates projectile mass to a target-based reference quantity, thereby providing a consistent basis for comparison across the investigated cases ^67,68^. It was more suitable than using the actual projectile volume, since the latter would introduce a more direct dependence on detailed projectile geometry, whereas the adopted form preserves a common reference length scale governed by the projectile diameter.

5 $${m}_{p}^{*}=\frac{{m}_{p}}{{\rho}_{d}{D}^{3}}$$

Since, ${D}_{r}$, is already defined as a percentage-based index, its dimensionless form (normalized sand relative density, ${D}_{r}^{*}$) follows naturally by converting it into a decimal scale between 0 and 1 (Eq. (6)), without altering its physical meaning as a descriptor of the sand state.

6 $${D}_{r}^{*}=\frac{{D}_{r}}{100}$$

The penetration depth was normalized by the projectile diameter as given in Eq. (7), so that the response could be interpreted in terms of penetration measured in projectile diameters.

7 $${d}_{p}^{*}=\frac{{d}_{p}}{D}$$

where (${d}_{p}^{*}$) is the normalized penetration depth. This normalization was adopted because the projectile diameter is the most relevant characteristic length for the local contact and penetration process, making the resulting expression more physically representative than scaling the depth by a less directly related geometric quantity. Accordingly, Table 5 presents the complete experimental results, including the actual and dimensionless values of the investigated parameters together with the measured and normalized penetration depths.

**Table 5:** Summary of the experimental results in actual and dimensionless form.

Test No	Variable	Actual results			Dimensionless results		
		Parameter value		*d*_*p*_ (mm)	Parameter value		*d*_p_*
T1	Repeatability tests *(Projectile of α* = *30°, against sand with D*_*r*_ = *95%)*	*–*		233	–		4.66
T2				231			4.62
T3				235			4.67
T4	Sand relative density	*D*_*r*_* (%*)	25	325	*D*_r_*	0.25	6.50
T5			55	197		0.55	3.94
T6			75	171		0.75	3.42
T7			95	162		0.95	3.24
T8	Projectile apex angle	*α* (°)	90	162	α*	1	3.24
T9			60	202		1.73	4.04
T10			30	235		3.73	4.70
T11			10	340		11.43	6.80
T12	Projectile mass	*m*_*p*_ (Kg)	2.445	162	*m*_p_*	10.98	3.24
T13			3.87	180		17.38	3.60
T14			5.33	189		23.94	3.78
T15			6.81	215		30.59	4.30

#### Effect of sand relative density (***D***_***r***_)

**Fig. 12:**
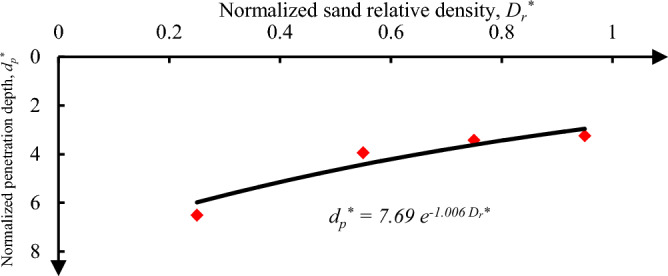
Effect of sand relative density on projectile penetration depth.

Figure 12 illustrates the dimensionless relationship between normalized sand relative density and the normalized penetration depth for tests *T4*-*T7* under identical test conditions. A clear inverse nonlinear trend is observed, where the normalized penetration depth decreases from 6.5 to 3.2 as the normalized relative density increases from 0.25 to 0.95. Relative to the loose sand condition, the normalized penetration depth at ${D}_{r}^{*}=0.55$, 0.75, and 0.95 is approximately 60%, 52%, and 49% of the corresponding value at ${D}_{r}^{*}=0.25$, respectively. This trend indicates that sand densification substantially increases penetration resistance. In loose sand, the relatively weak particle contact network allows easier particle rearrangement and localization displacement during impact, which facilitates deeper penetration. As the relative density increases, interparticle contact forces and confinement become stronger, Resulting in greater stiffness and higher resistance along the penetration path. Consequently, the denser granular skeleton more effectively restrains projectile intrusion and reduces the final normalized penetration depth, with *d*_p_^*^ at *D*_r_^*^ = 0.55, 0.75 and 0.95 decreasing to approximately 60%, 52%, and 49% of the corresponding value at the loose state of sand, i.e. *D*_r_^*^ = 0.25, respectively. This pronounced decrease further confirms the dominant role of sand densification in enhancing penetration resistance through increased interparticle interactions and confinement.

The experimentally obtained data exhibits nonlinear response, which is represented by an empirical exponential relationship (Eq. (8)) describing the influence of sand relative density on penetration depth under the same experimental constraints and boundary conditions.

8 $${d}_{p}^{*}=7.69{ e}^{-1.006 {D}_{r}^{*}}$$

#### Effect of projectile nose apex angle (${\boldsymbol{\alpha}}$)

**Fig. 13:**
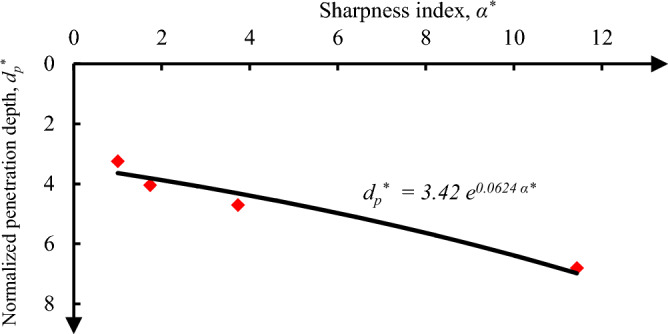
Dimensionless influence of projectile apex-angle parameter on normalized penetration depth for the experimental tests.

A series of free-fall penetration tests was conducted using conical projectiles with apex angles of 10°, 30°, 60°, and 90° to investigate the influence of projectile apex angle on penetration. All tests were performed on very dense sand (${D}_{r}=95\mathrm{\%}$) under identical boundary and loading conditions, including a constant drop height of 4.295 m. The experimentally measured penetration depths clearly demonstrated nonlinear dependence on the apex angle. To place this response on a unified comparative basis, Eq. (9) presents the dimensionless relationship between the apex-angle parameter (${\alpha }^{*}$) and the normalized penetration depth (${d}_{p}^{*}$). Because ${\alpha }^{*}$ increases as the conical tip becomes sharper, the trend appears direct and nonlinear in the dimensionless plot, although in terms of the actual apex angle the penetration response decreases as the nose becomes blunter. The normalized penetration depth increases from 3.2 at ${\alpha }^{*}$= 1 to 6.8 at ${\alpha }^{*}$= 11.43, indicating a marked increase in penetration as the nose geometry becomes sharper. The dimensionless experimental data are well represented by the exponential relation shown below and in Fig. 13.

9 $${d}_{p}^{*}=3.42 {e}^{0.624 {\alpha }^{*}}$$

From a physical standpoint, sharper nose geometries promote localized stress transfer at the soil–projectile interface, and reduce the effective contact area, thereby facilitating deeper penetration. By contrast, blunter noses distribute contact stresses over a wider area and mobilize greater lateral soil resistance, which limits penetration. When normalized relative to the sharpest configuration (*α* = 10°, α^*^ = 11.43 and ${d}_{p}^{*}$ = 6.8), the penetration depth decreases to approximately 69%, 59%, and 47% at *α* = 30°, 60°, and 90°, respectively. This progressive reduction confirms that blunter geometries increase penetration resistance, while the exponential form of the proposed relationship indicates that the sensitivity of the response becomes less pronounced as the projectile approaches blunt configurations.

#### Effect of projectile mass (***m***_***p***_)

**Fig. 14:**
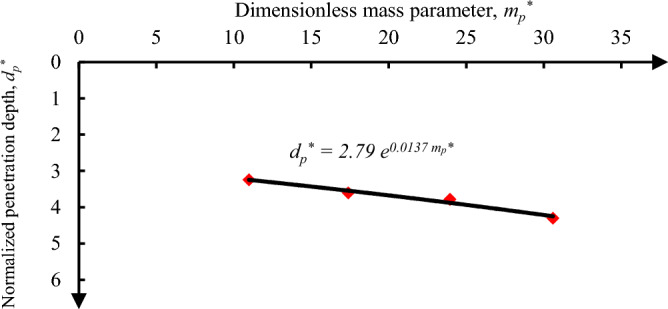
Dimensionless influence of projectile mass parameter on normalized penetration depth for the experimental tests.

The influence of varying the projectile mass on the penetration depth was investigated, while keeping the other controlling parameters with fixed values, ($\alpha$ = 90°, *D*_*r*_ = 95%, and *H* = 4.295 m). Four mass configurations ranging from 2.445 kg to 6.81 kg were examined using removable mass extensions. A direct nonlinear trend is observed (Eq. (10)), with ${d}_{p}^{*}$ increasing from 3.2 to 4.2 as ${m}_{p}^{*}$ increases from 10.98 to 30.59, as presented in Fig. 14. When referenced to the lightest projectile (*m*_*p*_ = 2.445 kg, *m*_p_^*^ = 10.98, and ${d}_{p}^{*}$ = 3.2), the normalized penetration depth increases progressively to approximately 113%, 119%, and 134% for projectile masses of 3.87 kg, 5.33 kg, and 6.81 kg, respectively. This trend confirms that increasing projectile mass enhances penetration capacity, although the response does not vary in simple linear proportion over the investigated range.

10 $${d}_{p}^{*} =2.79{ e}^{0.0137{ m}_{p}}$$

Physically, increasing projectile mass increases the inertia and impact energy available at contact, thereby promoting deeper penetration. At the same time, the nonlinear form of fitted relation indicates that the penetration response is governed not only by the increase in projectile mass itself, but also by the evolving interaction between projectile inertia and the resistance mobilized within the dense granular medium.

### Numerical results

Table 6 compiles the numerical results in both actual and dimensionless form which will deeply discussed in the following subsections with emphasis on the effects of projectile apex angle, projectile mass, sand internal friction angle, drop height, projectile diameter, projectile nose geometry, and impact angle on the penetration response and, where applicable, crater development. In addition to the previously defined parameters ${\alpha }^{*}$, ${m}_{p}^{*}$, ${D}_{r}^{*}$, and ${d}_{p}^{*}$, the remaining variables were also expressed in dimensionless form to maintain a unified basis for comparison across the numerical cases. For the internal friction angle, the adopted transformation is given in Eq. (11) as friction coefficient ($\mu$). This form was selected because, within the Mohr–Coulomb framework, $\mathrm{tan}\phi$ corresponds directly to the coefficient of internal friction^57,69^, and therefore represents the frictional contribution of the granular medium more meaningfully than the angle value alone.

**Table 6:** Summary of the numerical results in actual and dimensionless form.

Test No	Variable	Actual results				Dimensionless Results			
		Parameter value		*d*_*p*_ (mm)	*C*_*D*_ (mm)	Parameter value		*d*_p_*	*C*_D_*
M1	Projectile apex angle	*α* (°)	105	139.17	141.83	α*	0.77	2.78	2.84
M2			75	170.25	148.95		1.30	3.41	2.98
M3			45	217.99	133.18		2.41	4.36	2.66
M4			20	254.97	81.18		5.67	5.1	1.62
M5	Projectile mass	*m*_*p*_ (Kg)	8.265	264.11	179.44	*m*_p_*	37.13	5.28	3.59
M6			9.72	287.56	180.47		43.66	5.75	3.61
M7			11.175	312.96	184.4		50.20	6.26	3.69
M8			12.63	338.16	180.47		56.73	6.76	3.61
M9	Angle of internal friction	*ϕ* (°)	36	255.88	112.48	$\mu$	0.73	5.12	2.25
M10			37	244.1	112.48		0.75	4.88	2.25
M11			38	239.45	112.4		0.78	4.79	2.25
M12			39	238.1	112.07		0.81	4.76	2.24
M13			40	219.85	110.77		0.84	4.4	2.22
M14			41	215.76	110.22		0.87	4.31	2.2
M15			42	213.34	110.22		0.9	4.27	2.2
M16	Drop height	*H* (m)	4.295	161.67	148.95	H*	85.9	3.23	2.98
M17			10	237.55	208.7		200	4.75	4.17
M18			25	298.79	360.48		500	5.98	7.21
M19			50	344.15	367.48		1000	6.88	7.35
M20	Projectile diameter	*D* (mm)	25	286.83	64.53	E*	2129.9	11.47	2.58
M21			50	246.64	120.91		1064.95	4.93	2.42
M22			75	233.19	125.38		709.97	3.11	1.67
M23			100	227.04	125.72		532.47	2.27	1.26
M24	Projectile nose geometry	Conical	248.02	112.09	Conical	4.96	2.24		
M25		Ogival	265.41	96.39	Ogival	5.31	1.93		
M26		Hemispherical	234.82	101.31	Hemispherical	4.7	2.03		
M27		Parabolic	186.03	129.28	Parabolic	3.72	2.59		
M28		Blunt	133.69	125.18	Blunt	2.67	2.5		
M29	Impact angle	*θ* (°)	90	248.02	–	θ*	1	4.96	–
M30			80	234.75	–		0.98	4.69	–
M31			60	197.95	–		0.87	3.96	–
M32			45	172.78	–		0.71	3.46	–
M33			30	146.06	–		0.50	2.92	–

11 $$\mu =tan(\phi )$$

The drop height was transformed into a normalized drop height parameter (${H}^{*}$) thorough normalization by the projectile diameter using Eq. (12), using the same characteristic geometric scale already adopted for the normalized penetration depth. This preserves consistency between the governing variable and the normalized response.

12 $${H}^{*}=\frac{H}{D}$$

For the projectile diameter cases, however, the diameter was not introduced as a separate dimensionless variable of the form $D/{D}_{ref}$, since the projectile diameter already serves as the reference length used in normalizing both penetration depth and crater diameter. Instead, its influence was represented through the dimensionless impact energy parameter (${E}^{*}$) defined in Eq. (13). This expression follows directly from combining the mass and height normalizations, leading naturally to a ${D}^{4}$ term in the denominator, rather than introducing an arbitrary exponent. In this way, the diameter effect is captured through its energetic role in the penetration problem while preserving a nontrivial comparative form. The use of density-length based scaling and dimensionless impact-energy measures is also consistent with broader dimensional analysis and impact similarity frameworks reported in the literature^68,70^.

13 $${E}^{*}={m}_{p}^{*}{H}^{*}=\frac{{m}_{p}\hspace{0.17em}H}{{\rho}_{d}{D}^{4}}$$

The projectile nose geometry was retained as a categorical variable rather than converted into a single continuous dimensionless scalar. This choice was considered more appropriate because the investigated profiles represent discrete geometric classes and forcing them into one scalar descriptor at this stage would impose an artificial ordering that is not equally meaningful for all responses. For the impact angle investigation, the angle parameter was expressed as shown in Eq. (14) as dimensionless impact angle parameter (${\theta }^{*}$). Since the impact angle in the present study is measured from the target surface, $\mathrm{sin}\theta$ directly represents the fraction associated with the normal component of the impact direction, increasing from zero in the limiting grazing case to unity under normal impact. This makes it more physically appropriate here than $\mathrm{cos}\theta$, which would correspond to the tangential component under the adopted angle definition. Previous impact angle studies have likewise shown that key impact measures can scale with $\mathrm{sin}\theta$ or powers of $\mathrm{sin}\theta$ when the angle is referenced to the horizontal or target surface^71,72^.

14 $${\theta }^{*}=sin(\theta )$$

Finally, for cases in which a consistent crater diameter could be defined, the crater diameter was normalized by the projectile diameter according to Eq. (15) as normalized crater diameter (${D}_{c}^{*}$), so that crater development could be interpreted on the same geometric basis used for the penetration response.

15 $${D}_{c}^{*}=\frac{{D}_{c}}{D}$$

For the impact angle cases, crater diameter was not included because the resulting crater geometry was not sufficiently regular to justify a single directly comparable crater diameter value.

#### Validation

**Fig. 15:**
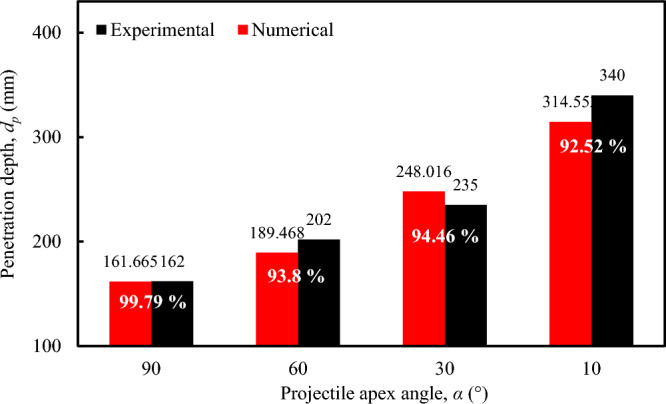
Numerical validation of penetration depth for projectiles with varying apex angles compared to experimental results.

The numerical study began with a validation phase to confirm the reliability of the proposed model. Four validation cases were considered using conical-nosed projectiles with apex angles of 90°, 60°, 30°, and 10°. These projectiles were numerically dropped onto very dense sand, with a relative density of 95%, from a fixed height of 4.295 m. The numerical penetration depths were compared with the experimentally obtained measurements, as presented in Table 7 and illustrated in Fig. 15. The numerical results showed good agreement with the experimental results, with validation ratios ranging from 92.52 to 99.79%, confirming that the proposed model reproduced the experimental penetration response with strong and satisfactory accuracy across the examined cases range.

**Table 7:** Numerical validation results.

Test No	Projectile apex angle, $\alpha$ (°)	Projectile mass, *m*_*p*_ (kg)	Penetration depth, *d*_*p*_ (mm)		Validation (%)
			Experimental	Numerical	
V1	10	3.665	340	314.55	92.52
V2	30	2.76	235	248.02	94.46
V3	60	2.48	202	189.47	93.8
V3	90	2.445	162	161.67	99.79

#### Effect of projectile nose apex angle (${\boldsymbol{\alpha}}$)

**Fig. 16:**
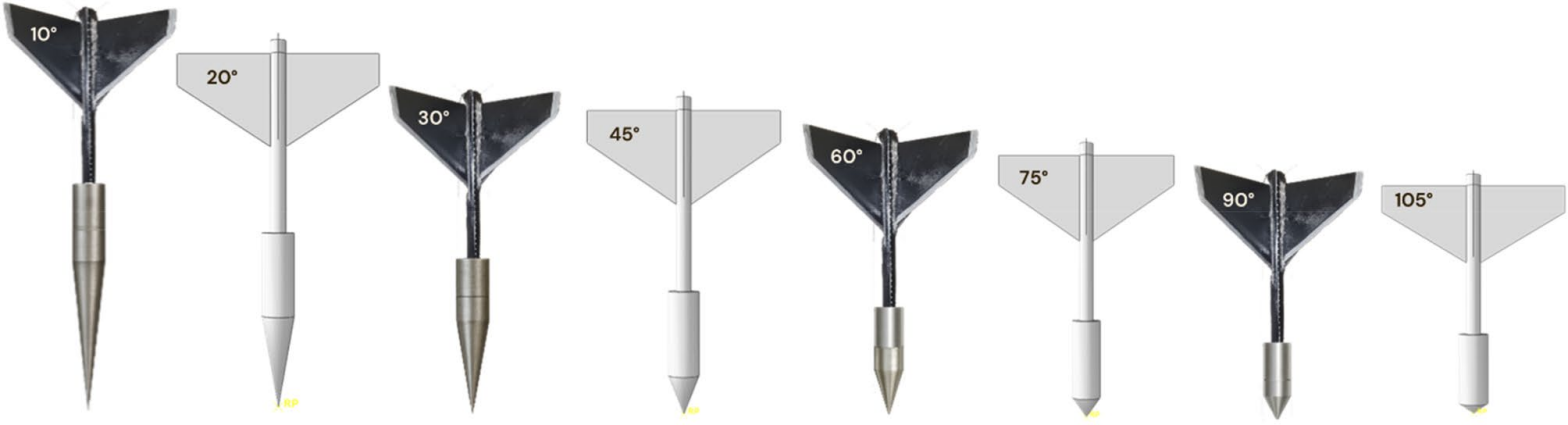
Physical and numerical conical nose projectiles of different apex angles.

To extend the experimental investigation and explore a wider range of nose geometries, the validated numerical framework was employed to simulate additional conical projectiles with apex angles of 20°, 45°, 75°, and 105°, while maintaining the same sand state, drop height, and boundary conditions (Fig. 16).

**Fig. 17:**
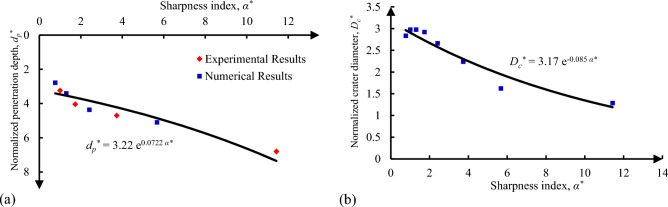
Dimensionless apex angle response of (**a**) normalized penetration depth and (**b**) normalized crater diameter.

Beyond expanding the investigated apex angle range, the additional numerical cases were incorporated with the experimental results to establish a more complete dimensionless dataset for the penetration response. In parallel, the validation-model simulations corresponding to the experimentally tested apex angles provided numerically obtained crater diameter values under the same loading and boundary conditions, thereby allowing the crater response to be examined on a consistent comparative basis for the apex angle cases. Accordingly, the combined dimensionless penetration depth results are presented in Fig. 17a, whereas the corresponding dimensionless crater diameter response is shown in Fig. 17b. The improved penetration depth trend is represented by the fitted relation in Eq. (16), which indicates and confirms that sharper noses promote deeper but more localized penetration, whereas blunter noses lead to shallower penetration accompanied by wider near-surface disturbance.

16 $${d}_{p}^{*}=3.22\hspace{0.17em}{e}^{0.0722{\alpha }^{*}}$$

The corresponding crater diameter relation is described by the second fitted relation (Eq. (17)), showing that ${D}_{c}^{*}$ decreases as ${\alpha }^{*}$ increases. This opposite response indicates that sharper noses promote deeper but more localized penetration, whereas blunter noses lead to shallower penetration accompanied by wider near-surface disturbance.

17 $${D}_{c}^{*}=3.166\hspace{0.17em}{e}^{-0.085{\alpha }^{*}}$$

Compared with the experimentally derived expression, the updated relation reflects the broader range of apex angles considered and the smoothing effect introduced by the numerical simulations and therefore provides a more complete representation of the apex angle effect. At the same time, the crater diameter relation was established from numerically obtained crater measurements for geometrically matched apex angle cases, enabling crater-related trends to be discussed within a consistent set of comparable conditions.

#### Effect of projectile mass (***m***_***p***_)

**Fig. 18:**
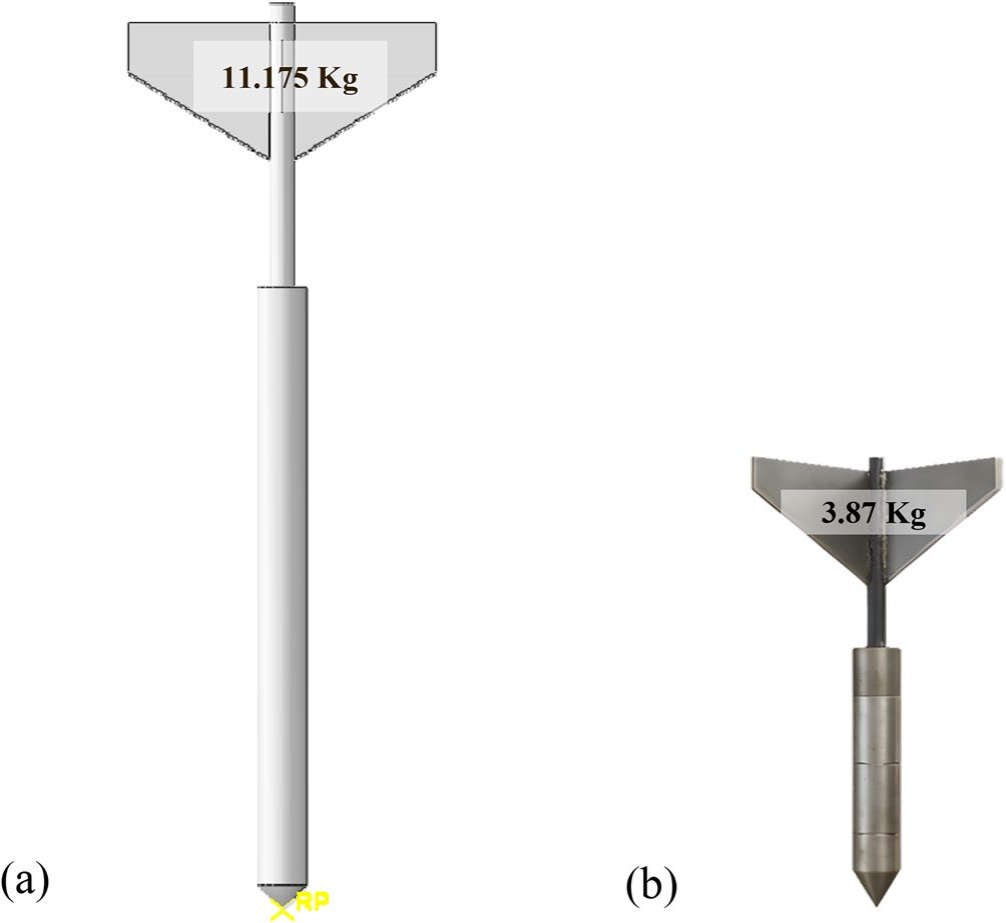
Visualization of projectile mass extensions. (**a**) Numerical projectile with five mass extensions. (**b**) Physical projectile with two mass extensions.

The numerical framework was further employed to extend the projectile mass investigation beyond the experimentally tested range. Four additional mass configurations were numerically established, namely 8.265 kg, 9.72 kg, 11.175 kg, and 12.63 kg corresponding to removable mass extensions of four, five, six, and seven units, under the same sand state, drop height, and boundary conditions. A representative comparison between the physical projectile and numerical projectile assemblies is provided in Fig. 18.

**Fig. 19:**
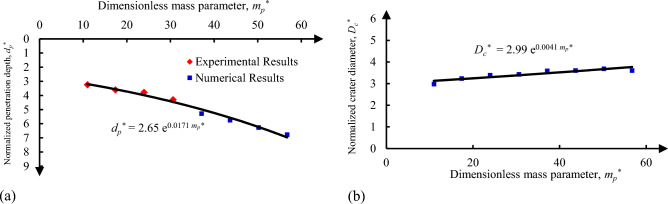
Dimensionless projectile mass response of (**a**) normalized penetration depth and (**b**) normalized crater diameter.

The combined dimensionless responses of penetration depth and crater diameter for the projectile mass series are presented in Fig. 19a and b, respectively. The fitted expressions describing both trends are given in Eq. (18) and Eq. (19).

18 $${d}_{p}^{*}=2.65\hspace{0.17em}{e}^{0.0171{ m}_{p}^{*}}$$

19 $${D}_{c}^{*}=2.99\hspace{0.17em}{e}^{0.0041{ m}_{p}^{*}}$$

The first relation shows that the normalized penetration depth increases nonlinearly with increasing normalized projectile mass. Over the combined experimental–numerical dataset, ${d}_{p}^{*}$ rises from 3.24 to 6.76 as ${m}_{p}^{*}$ increases from 10.98 to 56.73, indicating that heavier projectiles achieve progressively deeper penetration under the investigated conditions. The exponential form in Fig. 19a also suggests that the penetration response remains sensitive to projectile mass throughout the studied range. A different trend is observed for crater diameter. Although ${D}_{c}^{*}$ increases with ${m}_{p}^{*}$, the variation remains comparatively limited, ranging from about 2.98 to 3.69. This indicates that projectile mass exerts a stronger influence on subsurface penetration than on the lateral extent of the surface crater. For the experimentally investigated mass levels, the corresponding crater diameter values is provided numerically under the same controlled conditions, which enabled the crater response to be assessed within the same mass series without altering the comparison basis.

#### Effect of sand internal friction angle (*ϕ*)

**Fig. 20:**
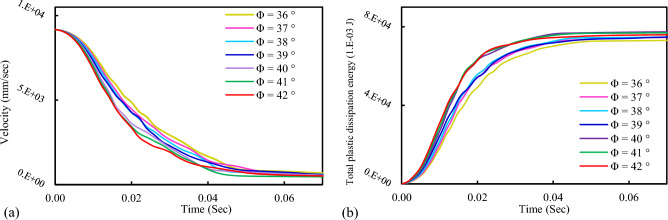
Effect of sand internal friction angle on projectile response. (**a**) Velocity–time history. (**b**) Total plastic dissipation energy evolution.

The internal friction angle of sand ($\phi$) is the primary parameter governing shear resistance in non-cohesive granular soils in accordance with the *MC* criterion^73,74^. For very dense sands, reported laboratory data indicate that $\phi$ typically ranges between 36° and 42°^75–78^. This range was therefore adopted for numerical parametric investigation, while all other soil and projectile parameters were kept constant to investigate the effect of varying the internal friction angle. The projectile velocity–time histories and total plastic dissipation energy evolutions shown in Fig. 20 reveal a consistent response pattern across the investigated friction angle range. In all cases, the response exhibits an initial rapid deceleration stage followed by a slower terminal stage as the projectile approaches rest. Immediately after impact, the velocity decreases sharply owing to the inertial resistance of the mobilized sand ahead of the nose and the rapid development of localized shear stresses. This is followed by a more gradual deceleration stage governed primarily by frictional resistance and quasi-static drag. The same trend is reflected in the plastic dissipation curves, where energy accumulates rapidly during the early penetration stage and then approaches a plateau as the projectile motion diminishes. This behavior is consistent with previous studies on projectile–granular interactions, where an initial inertial-dominated deceleration stage is followed by a friction-controlled, quasi-static phase as the penetrator slows to rest^5,9^.

**Fig. 21:**
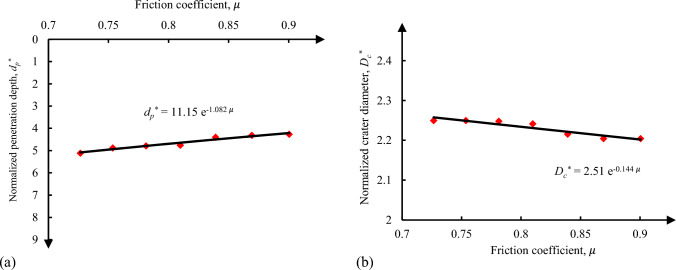
Dimensionless effect of sand internal friction angle on (**a**) normalized penetration depth, and (**b**) normalized crater diameter.

At lower internal friction angles, the reduced shear resistance allows increased particle mobility, enabling grains to slide and rearrange more freely around the projectile. As a result, the projectile maintains a relatively higher velocity over a larger portion of its penetration path, and its kinetic energy is dissipated more gradually through distributed plastic deformation. With increasing internal friction angle, particle interlocking becomes progressively more pronounced, leading to higher effective stresses and increased resistance to shear. This transition is reflected in a faster rate of velocity reduction and a more rapid accumulation of plastic dissipation energy. As the internal friction angle approaches $\phi ={42}^{\circ }$, the sand exhibits strong interlocking and dilative behavior, producing elevated stresses at the projectile tip and enhanced radial confinement. Under these conditions, the projectile experiences a higher deceleration rate, resulting in a shorter stopping time and a reduced penetration distance. This trend is further captured in the dimensionless response shown in Fig. 21, the normalized penetration depth decreases with increasing $\mu$, while the normalized crater diameter exhibits only a slight reduction over the same range.

The dimensionless penetration depth response is represented by the exponential correlation given in Eq. (20), while the corresponding crater diameter trend is described by Eq. (21).

20 $${d}_{p}^{*}=11.15 {e}^{-1.082\mu }$$

21 $${D}_{c}=2.51{ e}^{-0.144\mu }$$

The first relation indicates a clear inverse dependence of normalized penetration depth on the internal friction parameter, with ${d}_{p}^{*}$ decreasing from about 5.1 to 4.3 as $\mu$ increases from approximately 0.727 to 0.9. By contrast, the change in normalized crater diameter remains limited, with ${D}_{c}^{*}$ varying only slightly from about 2.25 to 2.20. This difference shows that, within the investigated range, the internal friction angle exerts a stronger influence on the depth of intrusion than on the lateral extent of the crater. Overall, increasing $\phi$ enhances the frictional and dilative resistance of the granular medium, thereby restricting penetration more noticeably than surface crater growth.

#### Effect of drop height (*H*)

**Fig. 22:**
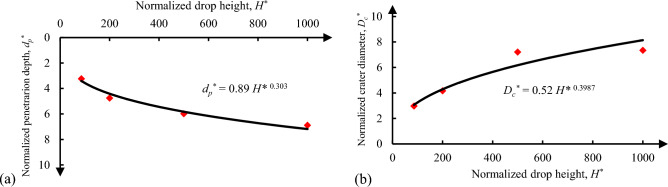
Projectile drop height effect on (**a**) penetration depth, and (**b**) crater diameter.

By changing the vertical drop height while keeping all other soil and projectile parameters constant, it was allowed to numerically investigate how drop height affects the soil-projectile interaction. Throughout the simulations, the sand was consistently kept at a relative density of 95%. The projectile featured a conical nose with an apex angle of 90° and weighed 2.445 kg. The drop height (*H*) varied from the experimentally determined 4.295 m to new heights of 10 m, 25 m, and 50 m. The drop height was transformed into impact velocity according to (Eq. (1)), resulting in equivalent impact speeds of 9.18 m/s, 14 m/s, 22.15 m/s, and 31.32 m/s for the four respective heights. The corresponding responses of normalized penetration depth and normalized crater diameter are shown in Fig. 22 and represented in Eqs. (22) and (23), respectively.

22 $${d}_{p}^{*}=0.89 {H}^{* 0.303}$$

23 $${D}_{c}=0.52 {H}^{* 0.3987}$$

Both normalized responses increase with increasing ${H}^{*}$, confirming that higher drop height increase the penetration process and enlarges the crater related response. Over the investigated range, ${d}_{p}^{*}$ increases from 3.2 at ${H}^{*}$ = 85.9 to 6.9 at ${H}^{*}$ = 1000, while ${D}_{c}^{*}$ rises from 3 to 7.3. The crater response, however, becomes less sensitive at the highest two levels, since the increase from 25 to 50 m is comparatively small relative to that observed at lower heights. This behavior suggests that, at the highest impact energies, part of the additional input is increasingly influenced by finite domain confinement and wave reflection effects rather than expressed solely as further crater enlargement.

**Fig. 23:**
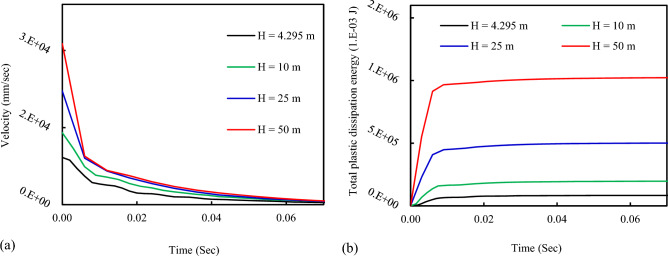
Effect of projectile drop height on the penetration process. (**a**) Velocity–time history. (**b**) Total plastic dissipation energy evolution.

Figure 23 illustrates how dynamic response changes across drop heights using projectile velocity and total plastic dissipation energy time histories. As the drop height increases, the projectile enters the soil with a higher initial velocity and undergoes a longer deceleration stage before approaching rest. At 4.295 m and 10 m, the projectile velocity decreases rapidly to near-zero values within a relatively shorter interval, while at 25 m and 50 m the deceleration process extends further in time. The total plastic dissipation energy follows the same ordering, increasing more rapidly and reaching substantially higher terminal values as the drop height increases. Together, these histories indicate that higher drop heights don’t merely increase the peak response but also extend the duration and soil-projectile interaction.

**Fig. 24:**
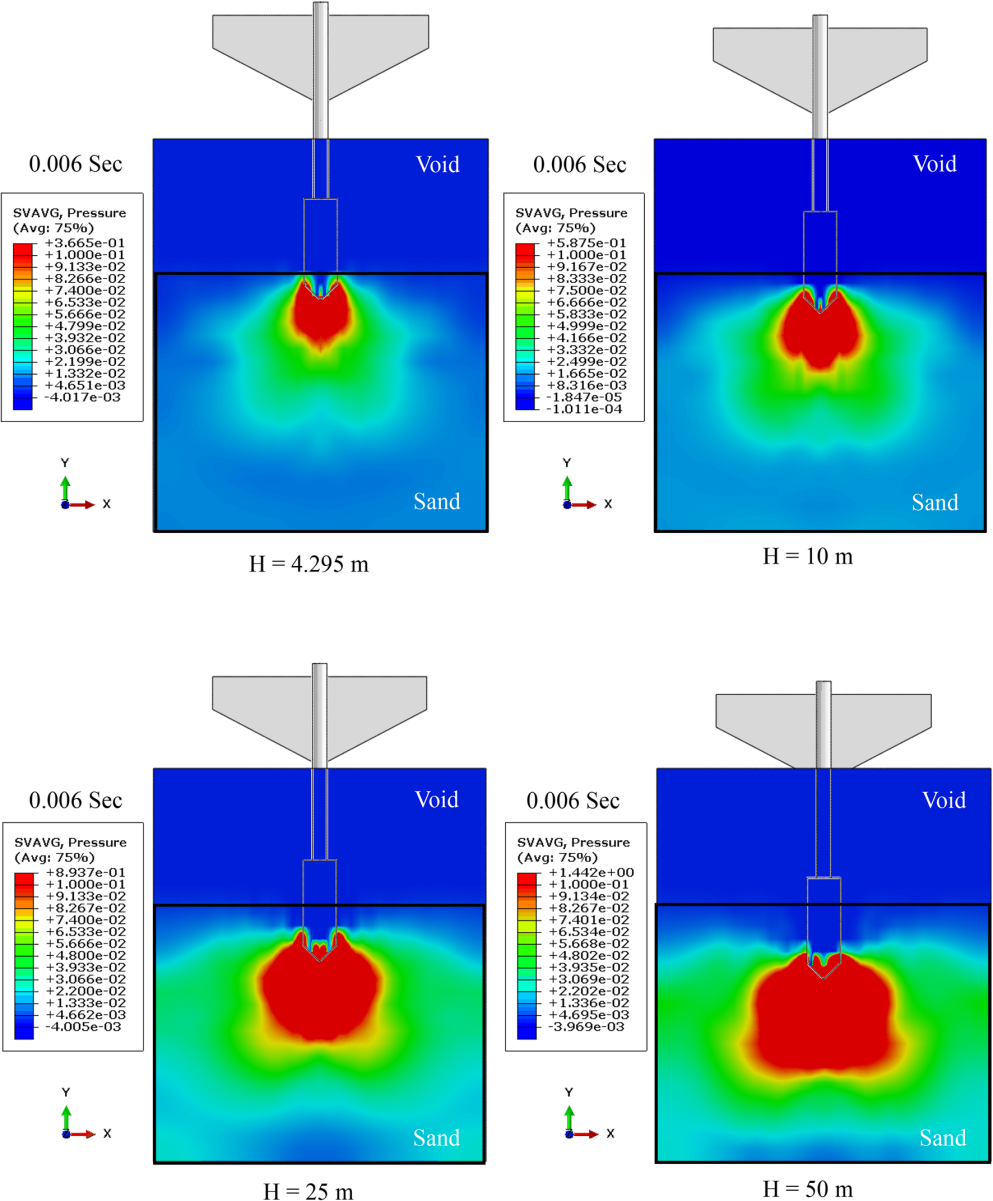
Stress for different drop heights at t = 0.006 s after impact.

**Fig. 25:**
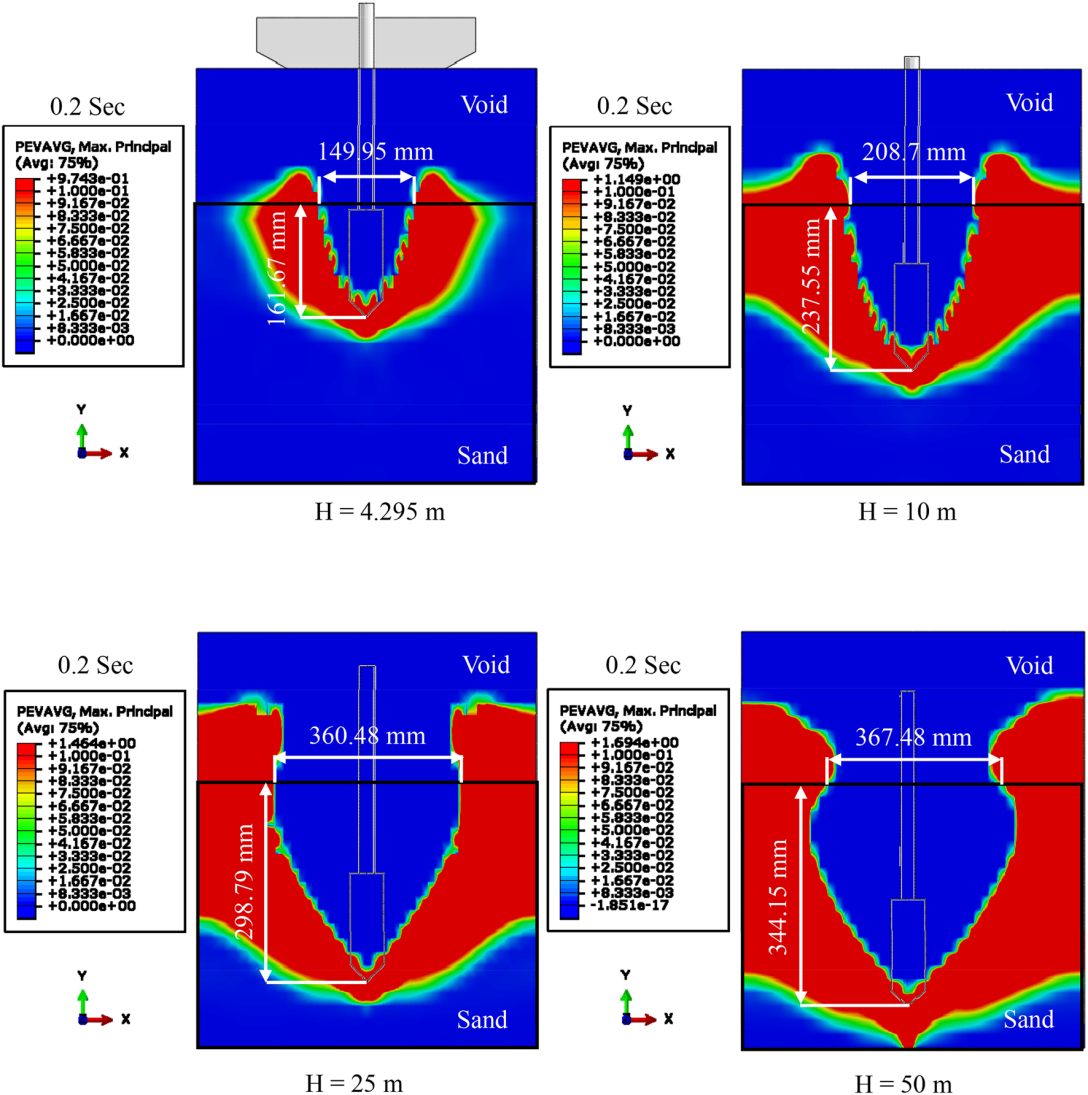
Total cumulative plastic strain for different drop heights at t = 0.2 s after impact.

The stress and cumulative average plastic strain contours from finite element analysis (Fig. 24 and Fig. 25) further clarify the drop height effect. At 4.295 m and 10 m, the high-stress zone remains concentrated around the projectile axis, generating a relatively narrow conical or cylindrical penetration core. At 25 m and 50 m, both the stress field and accumulated plastic strain spread more laterally and occupy a wider portion of the soil domain. This pattern indicates a transition focused penetration to broader deformation and stronger radial propagation of the induced waves. At higher drop heights, the laterally propagating stress waves approaches tank rigid boundaries and interact again with the central region penetration region after reflection. Thus, apparent penetration depths and crater dimensions no longer accurately represent unconfined or field-scale soil domain behavior. Then the diameter ratio criterion proposed by Seguin et al.^56^ is valid only if the diameter ratio is 10 and for relatively low impact velocities (up to roughly 10 m/s); beyond this range. Accordingly, the responses at the highest energy levels should be interpreted with appropriate consideration of finite domain effects, particularly for crater growth and the apparent extent of penetration-related deformation.

#### Effect of projectile diameter (*D*)

**Fig. 26:**
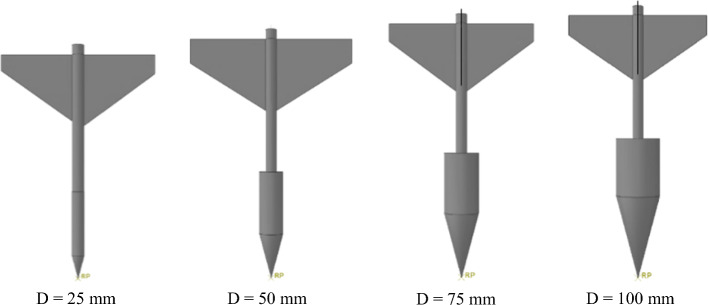
Projectiles of different diameters.

Four numerical simulations were conducted for the projectiles in this part of the study. These projectiles had a fixed nose apex angle (*α* = 30°), the same mass (*m*_*p*_ = 2.76 kg), and were released from the same drop height (*H* = 4.295 m) against a very dense sandy soil (*D*_*r*_ = 95%). As shown in Fig. 26, the projectile diameter (*D*) was systematically modified to represent values ranging from 25, 50, 75, and 100 mm. It is worth noting that this range was selected to ensure that the diameter ratio (*B/D*) is not less than 5, in accordance with the boundary conditions effect limitations and scaling recommendations reported by Seguin et al.^56^. It should be noted that, to change the diameter of the projectile and keep a constant apex angle, the length of the simulated projectiles was slightly increased, as presented in Fig. 26.

**Fig. 27:**
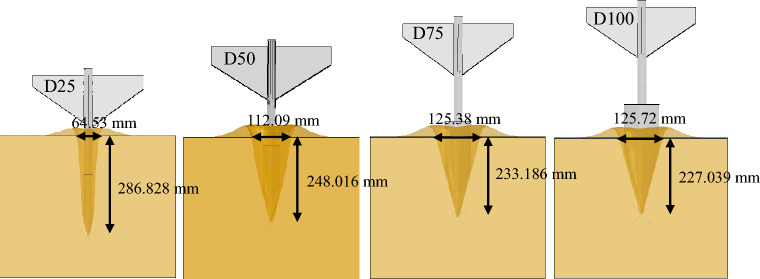
Penetration depths and crater diameters of the projectiles with different diameters.

**Fig. 28:**
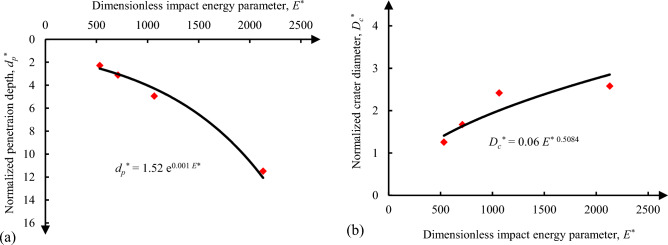
Effect of the diameter-related energy parameter on (**a**) normalized penetration depth, and (**b**) normalized crater diameter.

The final penetration and crater diameter measurements confirms that projectile diameter has a marked influence on the soil-projectile interaction. As illustrated in Fig. 27, reducing the projectile diameter from 100 to 25 mm increases the penetration depth from 227.04 to 286.83 mm, while the measured crater diameter decreases from 125.72 to 64.53 mm. For comparative interpretation, however, the diameter effect is expressed here through the dimensionless impact energy related parameter ${E}^{*}={m}_{p}^{*}{H}^{*}$, since the projectile diameter already serves as the reference length in both ${d}_{p}^{*}={d}_{p}/D$ and ${D}_{c}^{*}={D}_{c}/D$. The resulting dimensionless penetration depth and crater diameter trends are presented in Eqs. (24) and (25) from Fig. 28.

24 $${d}_{p}^{*}=1.52\hspace{0.17em}{e}^{0.0012{ E}^{*}}$$

25 $${D}_{c}^{*}=0.06\hspace{0.17em}{E}^{* 0.5084}$$

The first relation indicates that the normalized penetration depth increases sharply as ${E}^{*}$ increases, rising from 2.3 at ${E}^{*}$ = 532.47 to 11.5 at ${E}^{*}$ = 2129.9. This means that, under otherwise identical conditions, smaller-diameter projectiles penetrate more deeply when the response is measured relative to their own diameter. The second relation shows that the normalized crater diameter also increases with ${E}^{*}$, from about 1.3 to 2.6, although the variation is less pronounced than that observed for ${d}_{p}^{*}$. Accordingly, decreasing projectile diameter enhances both the relative penetration capacity and the relative crater size, even though the absolute crater diameter itself becomes smaller. This behaviour is physically consistent from the perspective of granular mechanics. Reducing projectile diameter decreases the frontal contact area and therefore increases the average contact pressure at the soil-projectile interface. Under the same mass and drop height, this promotes a more concentrated transfer of impact energy into the penetration depth, allowing the projectile to advance further before the available energy is dissipated. By contrast, larger-diameter projectiles mobilize resistance over a broader contact region, which limits penetration and promotes a wider but relatively shallower surface disturbance.

**Fig. 29:**
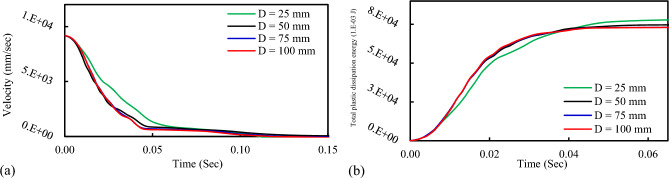
Effect of projectile diameter on the penetration process. (**a**) Velocity–time history. (**b**) Total plastic dissipation energy evolution.

The velocity–time histories in Fig. 29a demonstrate that larger-diameter projectiles decelerate more rapidly from the beginning of penetration and reach near-zero velocity in a shorter period than the smaller-diameter projectiles. This is consistent with the greater frictional and shear resistance mobilized along the penetration path as contact area increases. The total plastic dissipation energy in Fig. 29b exhibits the same general evaluation for all cases, with a rapid initial rise followed by a gradual approach to a terminal level. However, the smaller-diameter projectiles attain slightly higher terminal dissipation values and maintain motion over a longer interval, which agrees with their deeper penetration and larger relative crater response. Taking together, the velocity and energy histories indicate that the projectile diameter governs not only the final penetration and crater measures but also reorganizes the manner in which impact energy is redistributed between forward intrusion and lateral deformation in the surrounding sand.

#### Effect of projectile nose geometry

**Fig. 30:**
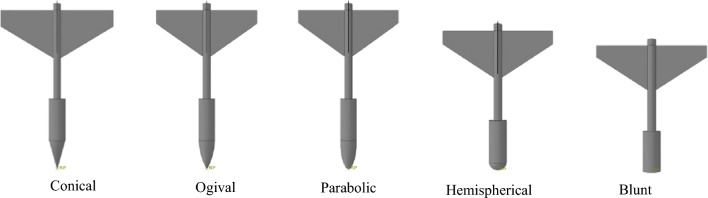
Visualization of projectiles with different nose geometries.

Projectile nose geometry is widely recognized as a governing parameter influencing penetration performance in granular and geomaterial targets. Previous experimental and numerical investigations have demonstrated that commonly adopted projectile nose configurations include conical, ogival, parabolic, hemispherical, and blunt geometries, each exhibiting distinct stress concentration patterns and energy transfer mechanisms during impact^2,32,33,79^. Based on these established classifications in the literature, five representative projectile nose geometries were selected for the present parametric investigation: conical with a 30° apex angle, ogival with a caliber radius head (CRH) of 3.7, parabolic with a ballistic length of 93 mm, hemispherical with a radius of 25 mm, and blunt, while maintaining all other variables constant, as illustrated in Fig. 30. Each projectile was modeled with a constant mass of 2.76 kg and a constant cylindrical diameter of 50 mm, dropped from a height of 4.295 m, resulting in an impact velocity of 9.18 m/s on very dense sand with a relative density of 95%.

**Fig. 31:**
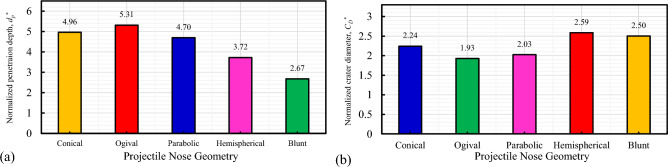
Projectile nose geometry effect on (**a**) normalized penetration depth, and (**b**) normalized crater diameter.

Figure 31 compares the normalized penetration depth and normalized crater diameter for the five nose geometries. Because projectile nose geometry is a categorical variable rather than a continuous scalar parameter, the response is discussed directly in dimensionless form rather than represented by a single fitted equation. The ogival nose produced the highest normalized penetration depth (${d}_{p}^{*}$ = 5.31), with the lowest normalized crater diameter (${C}_{D}^{*}$ = 1.93), indicating the most efficient penetration behavior among the investigated shapes. The conical and parabolic noses followed, with ${d}_{p}^{*}$ = 4.96 and 4.7, respectively, whereas the hemispherical and blunt noses gave the lowest normalized depths, 3.72 and 2.67, together with the largest crater responses. This trend is mechanically consistent with the manner in which each nose profile transfers load to the surrounding sand. The ogival nose promotes a smoother curve and more axial stress transfer, which limits early lateral grain displacement and allows deeper penetration with a comparatively narrow crater. The conical nose also performs efficiently, but its sharper flank geometry creates a steeper pressure ahead of the tip, causing stronger lateral grain deflection than in the ogival case.

**Fig. 32:**
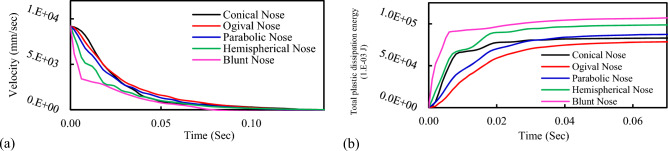
Effect of projectile nose geometry on the penetration process. (**a**) Velocity–time history. (**b**) Total plastic dissipation energy evolution.

Conversely, the hemispherical and blunt noses mobilize a broader frontal resistance over a larger contact area, leading to shallower penetration and a wider near-surface disturbance zone. The parabolic nose exhibits an intermediate response between the slender and blunt configurations. Figure 32 supports this interpretation through the projectile velocity–time histories and total plastic dissipation energy evolutions. The ogival and conical noses decelerate more gradually and maintain a higher residual velocity over a longer penetration interval, allowing deeper penetration and more efficient energy dissipation. whereas the hemispherical and blunt noses lose velocity more rapidly and accumulate plastic dissipation energy earlier in the impact event. The parabolic nose exhibits an intermediate response between the slender and blunt configurations.

**Fig. 33:**
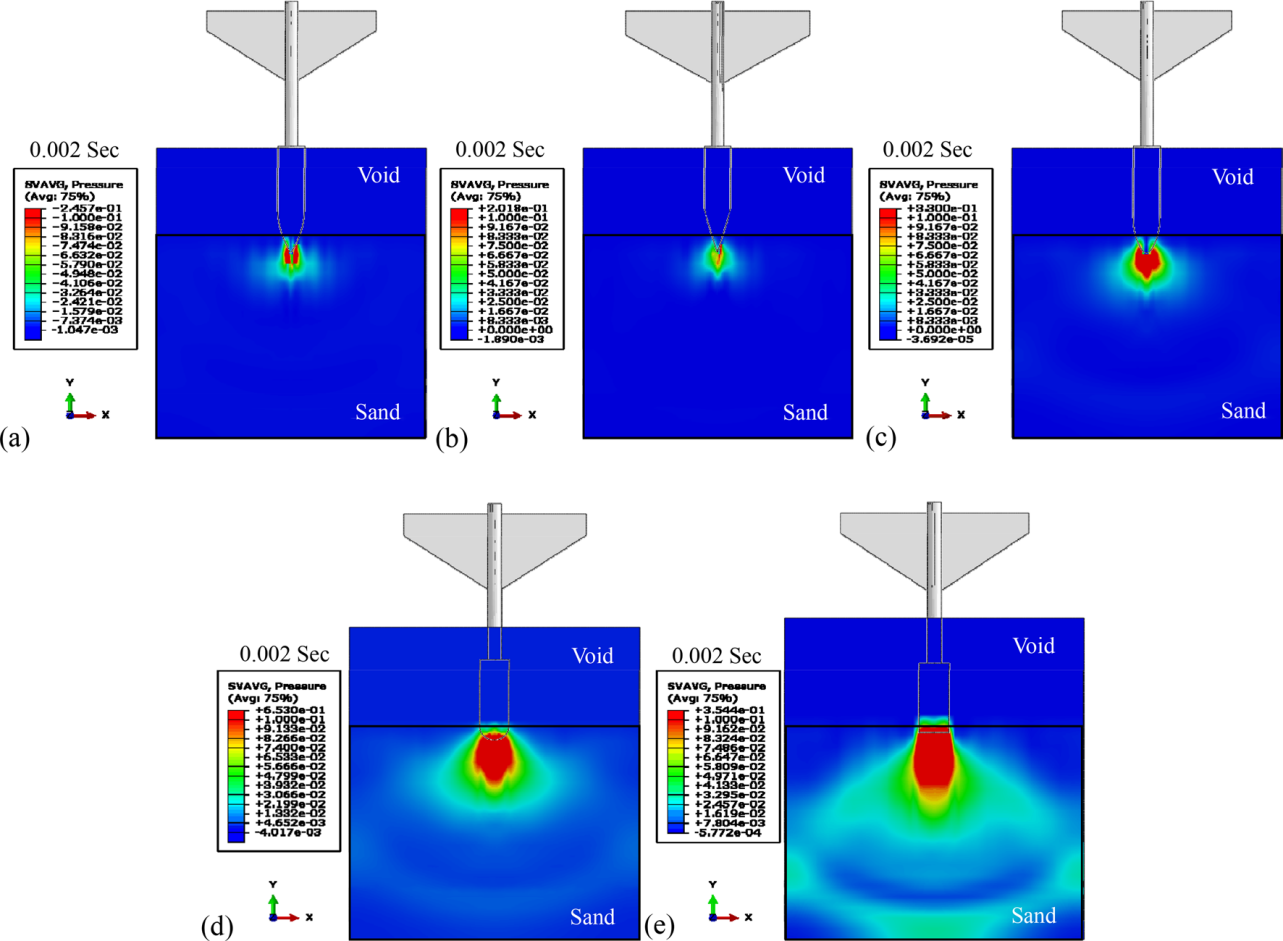
Stress wave contours at t = 0.002 s after impact for projectile with (**a**) conical, (**b**) ogival, (**c**) parabolic, (**d**) hemispherical, and (**e**) blunt nose shape.

Figure 33 further confirms these differences through the early stress wave contours. The ogival, conical, and parabolic noses generate more focused axial stress transmission beneath the projectile tip, while the hemispherical and blunt noses produce broader near-surface compression zones and stronger lateral spreading. These stress patterns explain why the slender nose geometries achieve deeper penetration, whereas the blunter shapes generate greater surface disturbance and larger normalized crater diameters.

#### Effect of projectile impact angle (*θ*)

**Fig. 34:**
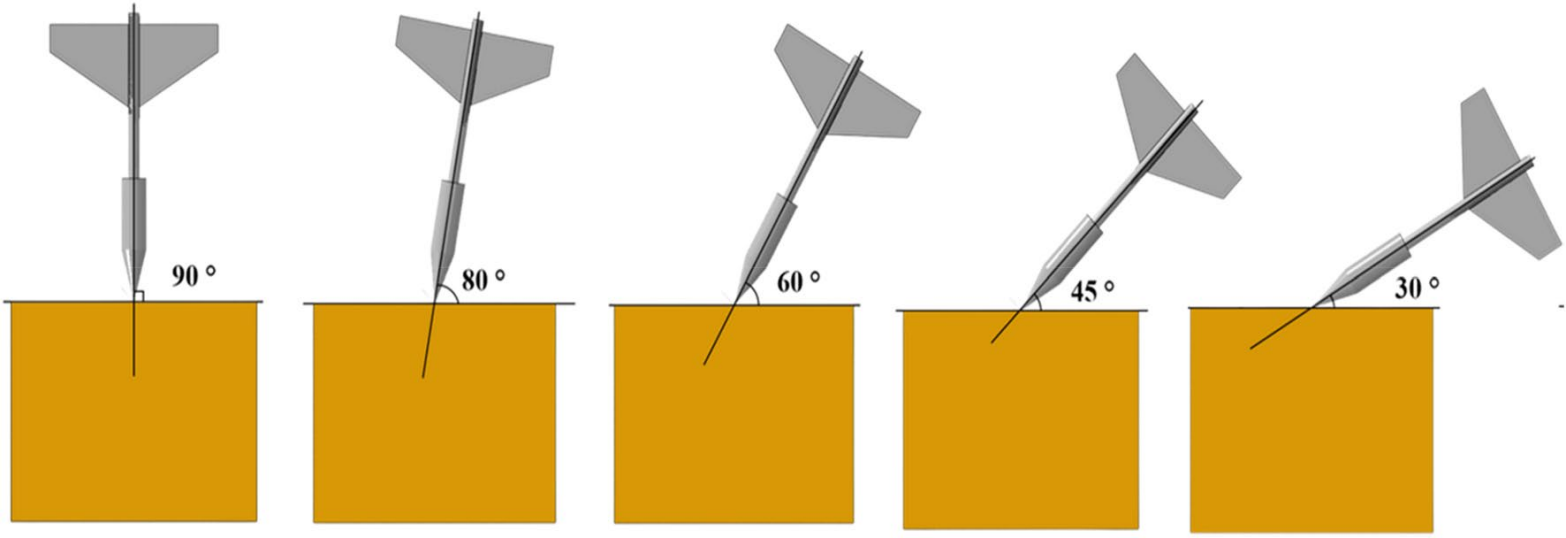
Oblique projectile impacts on sand at five impact angles (90°, 80°, 60°, 45°, 30°).

During free-fall, projectiles trajectories may deviate from ideal normal impact, leading to impact the sand surface with an impact angle rather than in a perfectly perpendicular manner. This effect was investigated numerically by varying the impact angle (*θ*) while keeping the remaining soil and projectile parameters constant. As shown in Fig. 34, five impact angles were considered namely 90°, 80°, 60°, 45°, and 30°, measured between the projectile axis and the sand surface.

**Fig. 35:**
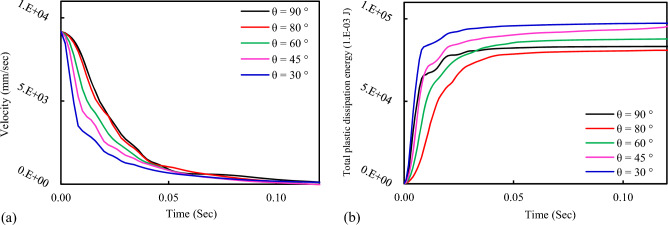
Effect of impact angle on the penetration process. (**a**) Velocity–time history. (**b**) Total plastic dissipation energy evolution.

The impact angle governs the partition of the projectile velocity into normal and tangential components and therefore directly affects the balance between penetration and surface sliding. Under normal impact (*θ* = 90°), the normal component reaches its maximum and the penetration response is correspondingly greatest. As the angle decreases. The tangential component becomes more pronounced, promoting near-surface sliding and redirecting a greater portion of the input energy into lateral deformation rather than deeper intrusion. Figure 35 supports this interpretation through the velocity–time history and total plastic dissipation energy history. Normal and near-normal cases maintain motion over a longer penetration interval, whereas the more oblique cases decelerate more rapidly after impact. The associated plastic dissipation histories also show that impact angle modifies not only the final penetration depth but also the rate and mode of energy transfer within the soil domain.

**Fig. 36:**
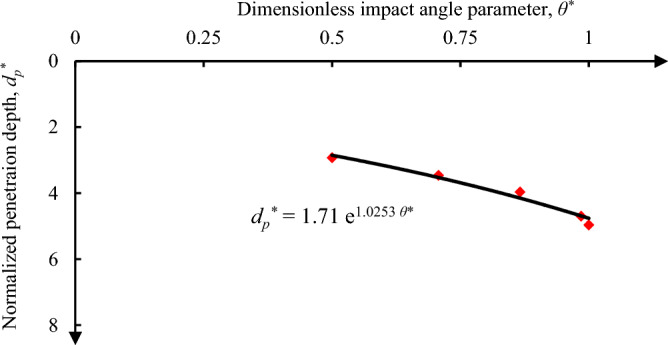
The resulted penetration depths plotted against different projectile impact angles.

The penetration response decreases progressively as the impact becomes more oblique. To capture this trend on a unified basis, the angular effect was expressed through the dimensionless parameter (θ^*^) This behavior is reflected directly in the penetration depth Eq. (26) represented in Fig. 36.

26 $${d}_{p}^{*}=1.71\hspace{0.17em}{e}^{1.023 {\theta }^{*}}$$

The fitted relation indicates a clear direct dependence of normalized penetration depth on ${\theta }^{*}$. Over the investigated range, ${d}_{p}^{*}$ decreases from 5 at ${\theta }^{*}$ =1 to 2.9 at ${\theta }^{*}$ = 0.5, confirming that penetration becomes progressively shallower as impact angle increases. This trend is consistent with the increasing diversion of momentum into tangential motion and near-surface shearing at lower angles. In the present study, crater diameter was not included in this comparison because oblique impact produced non-axisymmetric surface disturbance, making a single directly comparable crater-diameter measure less representative for this parameter study field.

### Sensitivity assessment

Following the experimental and numerical parametric results presented above, an additional sensitivity assessment was performed to consolidate the governing trends on a unified basis and to identify the most critical parameters influencing penetration depth and crater diameter within the investigated range. This assessment was built on the normalized response relations established in Sections “Experimental Results” and “Numerical Results” and was further extended through Spearman-based correlation mapping and response-span-based ranking. In this way, the analysis provides not only a comparative view of parameter-response associations, but also a more practical measure of the magnitude of variation induced by each parameter over the investigated range.

#### Correlation analysis

**Fig. 37:**
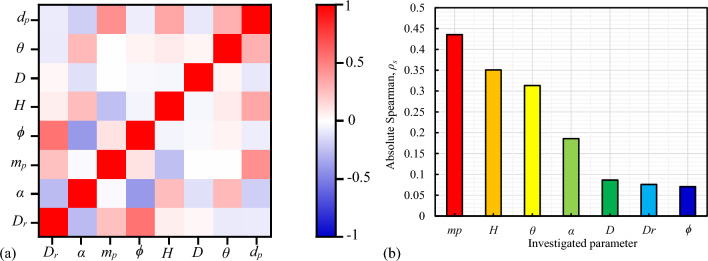
Spearman correlation analysis of penetration depth: (**a**) correlation heatmap and (**b**) parameter ranking by absolute Spearman coefficient.

To further interpret the normalized response relations established earlier, a correlation-based assessment was performed to examine the direction and relative consistency of the parameter response trends within the investigated range. For this purpose, Spearman’s rank correlation coefficient (${\rho}_{s}$) was adopted, since it provides a nonparametric measure of the strength and direction of monotonic association and is therefore more suitable for the present dataset, where the response trends are ordered but not necessarily linear^80,81^. The resulting Spearman correlation matrix for penetration depth is presented in Fig. 37a, while the corresponding absolute-value ranking is presented in Fig. 37b.

For the penetration depth response, the correlation pattern indicates that projectile mass exhibits the strongest monotonic association, followed by drop height and impact angle. The corresponding ${\rho}_{s}$ values are 0.435, 0.35, and 0.313, respectively, which indicates that increasing these parameters is generally associated with increasing penetration depth over the investigated range. By contrast, apex angle, projectile diameter, sand relative density, and internal friction angle show inverse associations with penetration depth, with apex angle producing the strongest inverse trend among them. The ranked correlation coefficients are summarized in Table 8, which provides a compact view of both the magnitude and direction of the observed monotonic associations.

**Table 8:** Spearman correlation summary and parameter ranking for penetration depth response.

Rank	Parameter	Spearman coefficient,${\rho}_{s}$	Absolute spearman coefficient,$\mid {\rho}_{s}\mid$	Direction
1	Projectile mass, *m*_*p*_ (Kg)	0.435	0.435	Direct
2	Drop height, *H* (m)	0.350	0.350	Direct
3	Impact Angle, $\theta$ (°)	0.313	0.313	Direct
4	Projectile apex angle, $\alpha$ (°)	−0.186	0.186	Inverse
5	Projectile diameter, *D* (mm)	−0.086	0.086	Inverse
6	Sand relative density, *D*_*r*_ (%)	−0.076	0.076	Inverse
7	Angle of internal friction, *ϕ* (°)	−0.071	0.071	Inverse

As mentioned earlier, the crater diameter assessment was restricted to parameter blocks for which a directly comparable scalar crater metric could be defined and to variables that could be represented on an ordered scale. Accordingly, the crater specific correlation matrix was limited to $\alpha$, ${m}_{p}$, $\phi$, $H$, and $D$, whereas sand relative density, projectile nose geometry, and impact angle were not carried forward into this part of the analysis. Table 9 summarizes the resulting coefficients and their directional ranking. Within this reduced set, the clearest monotonic signature is associated with apex angle, which shows a strong direct correlation with crater diameter. Drop height follows with a moderate positive association, whereas internal friction angle shows a comparable but inverse trend. Projectile mass remains distinctly weaker, and projectile diameter shows only a marginal positive association.

**Table 9:** Spearman correlation summary and parameter ranking for crater diameter response.

Rank	Parameter	Spearman coefficient,${\rho}_{s}$	Absolute spearman coefficient,$\mid {\rho}_{s}\mid$	Direction
1	Projectile apex angle, $\alpha$ (°)	0.893	0.893	Direct
2	Drop height, *H* (m)	0.482	0.482	Direct
3	Angle of internal friction, *ϕ* (°)	−0.47	0.469	Inverse
4	Projectile mass, *m*_*p*_ (Kg)	0.196	0.196	Direct
5	Projectile diameter, *D* (mm)	0.058	0.058	Direct

**Fig. 38:**
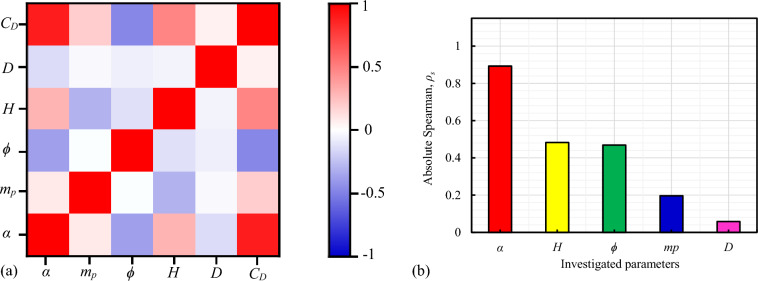
Spearman correlation analysis of crater diameter: (**a**) correlation heatmap and (**b**) parameter ranking by absolute Spearman coefficient.

This contrast becomes more evident in Fig. 38. Unlike the penetration-depth response, the crater-diameter structure is more sharply differentiated, with $\alpha$ emerging as the dominant monotonic parameter by a clear margin. The correlation analysis therefore clarifies the direction and relative consistency of the observed parameter-response trends; however, it was not used on its own to identify the most critical parameter. That final judgment was reserved for the normalized response-span analysis, since monotonic association alone does not necessarily reflect the practical magnitude of variation over the investigated range.

#### Sensitivity ranking

**Fig. 39:**
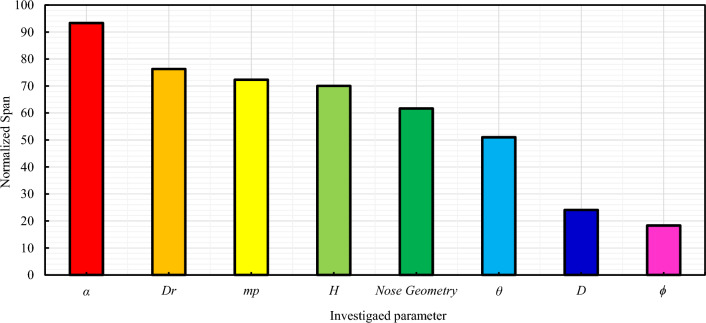
Sensitivity ranking of the investigated parameters for penetration depth based on normalized response span.

While the previous correlation analysis clarified the direction and monotonic consistency of the parameter-response trends, a separate ranking step was required to determine which parameters produced the critical practical variation in the responses over the investigated intervals. For this purpose, the present study adopted the normalized response span, calculated for each parameter as the difference between the maximum and minimum response values divided by the average response of that same results range and expressed as a percentage. In this form, the ranking reflects the relative magnitude of change induced by each parameter rather than the consistency of its monotonic trend alone. The penetration depth hierarchy obtained from this procedure is summarized in Table 10. The order shows that the apex angle produced the largest normalized span, reaching 93.33%, followed by sand relative density, projectile mass, and drop height. Nose geometry presented an intermediate contribution, presenting a non-monotonic substantial span of 61.67, where changing the geometry of the nose controlled the response independently, and the influence of each of the investigated nose geometries is not comparable. On the other hand, impact angle, projectile diameter, and internal friction angle showed progressively lower influence levels. This ranking indicates that the penetration response is critically influenced by both apex angle and soil’s relative density. Figure 39 shows sensitivity ranking of the investigated parameters for penetration depth.

**Table 10:** Normalized response span ranking for penetration depth response.

Rank	Parameter	*d*_*p,max*_ (mm)	*d*_*p,min*_ (mm)	Response span (mm)	Normalized span	Direction
1	Projectile apex angle, $\alpha$ (°)	340	139.17	200.83	93.33	Inverse
2	Sand relative density, *D*_*r*_ (%)	325	162	163	76.26	Inverse
3	Projectile mass, *m*_*p*_ (Kg)	338.16	162	176.16	72.32	Direct
4	Drop height, *H* (m)	344.147	161.67	182.48	70.04	Direct
5	Projectile nose geometry	265.409	133.69	131.72	61.67	Non-monotonic
6	Impact angle, $\theta$ (°)	248.016	146.06	101.96	51	Direct
7	Projectile diameter, *D* (mm)	286.828	227.04	59.79	24.07	Inverse
8	Angle of internal friction, *ϕ* (°)	255.876	213.34	42.54	18.31	Inverse

**Fig. 40:**
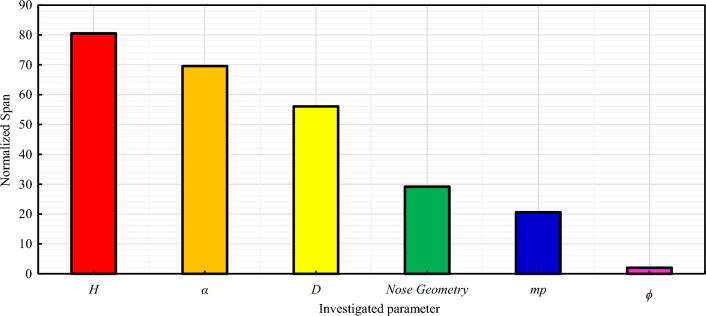
Sensitivity ranking of the investigated parameters for crater diameter based on normalized response span.

Crater diameter on the other hand presented different sensitivity ranking. The largest normalized span is associated with drop height, which reached 80.52%, followed by apex angle and projectile diameter. Nose geometry remains a secondary level of effect, while projectile mass and internal friction angle presented minor contribution on crater diameter variation, as presented in Table 11. In consistence with the crater-response scope established earlier, this ranking was restricted to parameters for which a directly comparable crater measuring was available over the investigated range. Figure 40 shows sensitivity ranking of the investigated parameters for crater diameter response.

**Table 11:** Normalized response span ranking for crater diameter response.

Rank	Parameter	*C*_*D,max*_ (mm)	*C*_*D,min*_ (mm)	Response span (mm)	Normalized span	Direction
1	Drop height, *H* (m)	367.48	148.95	218.53	80.52	Direct
2	Projectile apex angle, $\alpha$ (°)	148.95	64.37	84.58	69.6	Direct
3	Projectile diameter, *D* (mm)	125.72	64.53	61.19	56.07	Direct
4	Projectile nose geometry	129.28	96.39	32.89	29.15	Non-monotonic
5	Projectile mass, *m*_*p*_ (Kg)	184.4	148.95	35.45	20.61	Direct
6	Angle of internal friction, *ϕ* (°)	112.48	110.22	2.26	2.03	Inverse

In general, it should be noted that by comparing the outcomes of Spearman correlation ranking and normalized span sensitivity ranking, the normalized-span results showed reliable basis for determining the critical parameters influencing both the penetration depth and the crater diameter. For penetration depth, the most critical parameter within the investigated range was the projectile apex angle, followed by sand relative density and projectile mass. For crater diameter, the dominant parameter was the drop height, followed by apex angle and projectile diameter. Accordingly, the ranking analysis complements the correlation results by converting the observed trends into a clearer statement of practical influence, which is the basis adopted in this study for the final identification of the most critical parameters.

The interpretation of the present results should be considered within the investigated low-velocity range and the specific experimental–numerical conditions adopted in this study. In addition, crater-related comparisons were limited to cases for which a directly comparable crater measure could be defined. For the highest drop height cases, the observed response should also be interpreted with appropriate consideration of finite domain effects, since wave reflection and boundary confinement may increasingly influence the measured penetration and crater-related behavior as the impact energy rises. Accordingly, the present findings provide a validated and systematic basis for low-velocity projectile penetration in granular sandy soils within the investigated range, while extension beyond these conditions should be undertaken with caution.

## Conclusions

This study presents a comprehensive parametric investigation into the behavior of granular sandy soils subjected to low-velocity projectile penetration, integrating systematic experimental testing with advanced numerical modeling via the Coupled Eulerian–Lagrangian (*CEL*) approach in Abaqus. The results demonstrate strong agreement between experimental and numerical findings, validating the reliability of the adopted *CEL* model for capturing the complex soil-projectile interaction. To strengthen the physical interpretation of the results, the study was further extended through dimensionless analysis, Spearman-based correlation mapping, and normalized response-span sensitivity ranking. The study’s primary findings are summarized as follows:

- Penetration depth depended mainly on how effectively the projectile could transfer its momentum and impact energy into the sand before that energy was dissipated or redirected laterally. Within the investigated conditions, penetration depth decreased with increasing sand relative density, internal friction angle, projectile diameter, and projectile apex angle, whereas it increased with increasing projectile mass and drop height. It also became progressively smaller as the impact deviated from the normal direction. Among the investigated nose profiles, the ogival geometry showed the highest penetration efficiency, while the hemispherical and blunt geometries produced the shallowest penetration response.
- The crater diameter response depended mainly on parameters that determine how far the energy spreads around the impact point. Results revealed that crater diameter increased markedly with increasing drop height and projectile diameter, whereas its sensitivity to internal friction angle remained comparatively limited over the investigated range. The ogival nose produced the narrowest crater response, while the hemispherical and blunt geometries generated broader near-surface disturbance.
- The findings specifically reveal that the diameter ratio criterion (*B/D* ≥ 5) established by Seguin et al.^56^ is only valid under quasi-static or very low-velocity impact conditions (impact velocities up to approximately 10 m/s). At higher velocities, reflected stress waves and boundary effects significantly distort penetration and crater measurements, limiting the applicability of this criterion to low-energy regimes.
- The sensitivity assessment clarified both the direction of the parameter-response trends and the practical magnitude of their effects. The Spearman-based correlation analysis was used to identify the consistency and direction of the monotonic associations, whereas the normalized response span was used to determine which parameters produced the largest variation over the investigated range. On this basis, the most critical parameter controlling penetration depth was the projectile apex angle, which showed an inverse effect, followed by sand relative density with an inverse effect and projectile mass with a direct effect. For crater diameter, the dominant parameter was the drop height with a direct effect, followed by projectile apex angle and projectile diameter, both of which also showed direct effects within the investigated comparable cases.

These insights provide both a fundamental understanding of the controlling mechanisms in soil-projectile interactions at low velocities. The investigation also identifies the critical parameters that strongly control penetration depth and crater development within the investigated conditions. Future research should focus on expanding the parameter space to include soil saturation effects, intermediate and higher impact velocities, and soil improvements techniques (reinforcements, layer replacements, etc.) to further enhance the predictive capabilities of soil–projectile interaction models.

## Data Availability

All data generated or analyzed during this study are included in this published article. Additional raw data and numerical simulation files are available from the first author, Abdul-Rahman K. Osman, upon reasonable request.

## Abbreviations

v: Impact velocity of the projectile
*H*: Drop height of the projectile
*H*^***^: Normalized drop height parameter
*g*: Gravitational acceleration
*d*_*p*_: Penetration depth of the projectile
*d*_*p*_^***^: Normalized penetration depth parameter
*D*: Projectile diameter
*l*_*b*_: Ballistic length
*D*_*c*_: Surface crater diameter
*D*_*c*_^***^: Normalized crater diameter parameter
*m*_*p*_: Total projectile mass
*m*_*p*_^***^: Dimensionless projectile mass parameter
*α*: Projectile nose apex angle
*α*^***^: Dimensionless projectile apex-angle parameter representing nose sharpness
*θ*: Projectile impact angle relative to soil surface
*θ*^***^: Dimensionless impact angle parameter
*D*_*r*_: Relative density of sand
*D*_*r*_^***^: Normalized sand relative density
*ϕ*: Internal friction angle of sand
*ϕ*_*peak*_: Peak internal friction angle of sand
*ϕ*_*critical*_: Critical internal friction angle of sand
$$\mu$$: Friction coefficient
$$\psi$$: Dilation angle
*c*: Soil cohesion
*c*_*0*_: Initial apparent cohesion
*c*_*r*_: Residual apparent cohesion
${h}_{c}$: Cohesion softening modulus
*E*: Young’s modulus of elasticity
*E*^***^: Dimensionless impact energy parameter
*ν*: Poisson’s ratio
*σ*_*n*_: Normal stress
*σ*_*1*_*,σ*_*2*_*,σ*_*3*_: Principal stresses
*σ*_1_^′^,*σ*_2_^′^,*σ*_3_^′^: Effective principal stresses
${\rho}_{d}$: Dry unit mass of sand
${\rho}_{d,max}$: Maximum dry unit mass of sand
${\rho}_{d,min}$: Minimum dry unit mass of sand
*e*: Void ratio of sand
*e*_*max*_: Maximum void ratio of sand
*e*_*min*_: Minimum void ratio of sand
*OMC*: Optimum moisture content
*C*_*c*_: Soil coefficient of curvature
*D*_*10*_: Effective grain size
*D*_*30*_: Grain size at 30% passing
*D*_*60*_: Grain size at 60% passing
*G*_*s*_: Specific gravity
*B*: Internal width of the testing tank
*B/D*: Tank internal width to projectile diameter ratio
*KE*: Kinetic energy of the projectile
*PE*_*d*_: Plastic dissipation energy in soil
*t*: Time
*CRH*: Caliber radius head (for ogival nose)
*EC3D8*: 8-Node Eulerian brick element
*EC3D8R*: 8-Node Eulerian brick element, reduced integration
*R3D4*: 4-Node rigid shell element
*CEL*: Coupled Eulerian–Lagrangian
*SPH*: Smoothed particle hydrodynamics
*MC*: Mohr–Coulomb constitutive model
*DP*: Drucker–Prager constitutive model
*AR*: Aspect ratio
*ES*: Element Size
${\rho}_{s}$: Spearman correlation coefficient
